# Investigation of the Mechanism of Action of Liu Miao San in the Treatment of Lipid Metabolism Disorders on the basis of Network Analysis and Dynamic Ultrasound

**DOI:** 10.2174/0118715303417569251201075052

**Published:** 2026-05-21

**Authors:** Yu-Jia Xu, Hua Hou, Lin Liu, Fang-Jie Bi

**Affiliations:** 1 Department of Echocardiography, Zibo Central Hospital, No. 54 Zhangdian District - Communist Youth League West Road, Zibo, 255000, Shandong Province, People’s Republic of China;; 2 Department of Heart Center, Zibo Central Hospital, No. 54 Zhangdian District - Communist Youth League West Road, Zibo, 255000, Shandong Province, People’s Republic of China

**Keywords:** Lipid metabolism disorders, Liu Miao San, network analysis, ultrasound, fatty liver, herbal formulations

## Abstract

**Introduction:**

Lipid metabolism disorders are a critical global health issue. Liu Miao San (LMS), a traditional Chinese medicine (TCM) formula, has demonstrated clinical efficacy against these disorders; however, its underlying mechanism remains unclear.

**Methods:**

Network analysis was performed to predict LMS targets using the TCMSP and ETCM databases, followed by protein–protein interaction (PPI) network construction and GO/KEGG enrichment analyses. These predictions were validated *in vitro* using oleic acid-stimulated HepG2 cells by assessing lipid accumulation (Oil Red O staining, TG/TC measurements) and SREBP1/PPARγ localization *via* immunofluorescence. *In vivo*, high-fat diet (HFD)-induced rat models were used. Rats underwent dynamic ultrasound assessments, plasma and liver biochemical analyses (TG, TC, LDL-C, HDL-C, ALT, AST, TNF-α, IL-6), H&E staining, and liver immunofluorescence.

**Results:**

Network analysis identified SREBP1 and PPARγ as core targets. *In vitro*, LMS reduced lipid accumulation, decreased TG/TC levels, inhibited SREBP1 nuclear translocation, and promoted PPARγ nuclear translocation. *In vivo*, LMS alleviated HFD-induced lipid disorders, improved liver histopathology, reduced plasma TG/TC/LDL-C/ALT/AST and hepatic TG/TC/TNF-α/IL-6 levels, and normalized ultrasound parameters (*e.g.*, liver volume and echogenicity). Immunofluorescence further confirmed SREBP1 inhibition and PPARγ activation in liver tissue.

**Discussion:**

LMS ameliorates lipid metabolism disorders through a dual regulatory mechanism: suppressing SREBP1 (reducing fatty acid and cholesterol synthesis) and activating PPARγ (promoting cholesterol efflux and exerting anti-inflammatory effects). This multitarget action addresses both hepatic and systemic lipid dysregulation.

**Conclusion:**

LMS exerts therapeutic effects *via* SREBP1/PPARγ modulation, supporting its clinical application in TCM and providing a methodological framework for mechanistic studies of complex herbal formulations.

## 1 INTRODUCTION

The term “lipid metabolism disorders” refers to abnormalities in the composition and quantity of lipids and their metabolites in blood and other tissues and organs, caused by congenital or acquired factors [1-3]. As living habits and diets have changed, the incidence of lipid metabolism disorders has increased significantly, with these disorders affecting one quarter of the world's population [4-7]. Lipid metabolism disorders can cause a series of diseases, such as hyperlipidemia and fatty liver, resulting in a decreased quality of life and an increased economic burden for patients [8-12]. Lipid metabolism disorders are gradually becoming a worldwide public health issue. Blood and liver lipid metabolism disorders often occur simultaneously, but currently, there are no drugs that can simultaneously target these two tissues [13-16]. The single-target treatments for lipid metabolism disorders are limited. For example, statins reduce hyperlipidemia but have no significant effect on fatty liver disease [17-21]. The general trend in the treatment of lipid metabolism disorders is the use of multitarget drugs.

The approaches used in traditional Chinese medicine (TCM) for the treatment of chronic metabolic diseases have multiple targets, good efficacy, and few side effects, and have received global attention [22]. Liu Miao San (LMS) is composed of six Chinese medicinal herbs: *Crataegus pinnatifida*, *Pseudocydonia sinensis*, *Hovenia dulcis*, *Poria cocos*, *Monascus purpureus*, and *Ganoderma lucidum*. With reference to the “Treatise on Febrile Diseases” and according to the theory of “five circuits and six qi” in TCM, TCMs are prepared so that their ingredients complement each other and exhibit synergistic therapeutic effects, resulting in significant clinical benefits [23-33]. However, the mechanisms of action and regulatory targets of LMS in the treatment of lipid metabolism disorders remain unclear, and the underlying therapeutic pathways warrant further study.

Network analysis is a method based on the concept of an active ingredient target–disease target network to predict and explain the mechanisms of action of TCMs in the treatment of relevant diseases [34, 35]. Network analysis *via* multiple databases can be used to investigate the interactions between various active metabolites of LMS and disease targets to identify potential therapeutic targets and related signaling pathways. This research strategy provides a feasible solution for studying the associations between various active metabolites of a TCM and disease targets, as well as a scientific theoretical basis for the study of the mechanism of action of LMS [36].

In this study, network analysis methods were first used to predict potential LMS targets and to analyze its therapeutic mechanisms. The predicted targets and mechanisms were then validated at both the cellular and animal levels using biological experimental techniques, including immunofluorescence staining. The findings confirmed the potential mechanism of action of LMS in lipid metabolism disorders, provided a theoretical scientific basis for its clinical application, and offered research insights for studying the pharmacodynamic mechanisms of TCMs [37].

## 2 MATERIALS AND METHODS

### 2.1 Animals

Eighteen male specific pathogen-free Sprague Dawley (SD) rats (6-8 weeks old, 220 ± 10 g) were purchased from the Shandong Animal Experiment Center (Animal Production License Number: SCXK 2014-0007). The rats were housed at an appropriate temperature (23 ± 5°C), and throughout the experiment they had free access to food and water. The detailed composition of the animal feed used in the study is provided in Table **1**.

### 2.2 Drugs

LMS consists of six Chinese medicinal herbs: *P. sinensis, C. pinnatifida, H. dulcis, M. purpureus, P. cocos,* and *G. lucidum*. For this study, these herbs were mixed at a mass ratio of 20:10:9:10:15:10, then ground and sieved. The powders were thoroughly mixed and boiled at a solid-to-liquid ratio of 1:10 for 2 hours. After standing, the supernatant was collected. This extraction process was repeated three times, and the combined supernatants were used to obtain the LMS stock solution. LMS powder was prepared by rotary evaporation of the stock solution followed by freeze-drying under vacuum.

### 2.3 Instruments

A BIO-RAD680 microplate reader (Bio-Rad, USA), vacuum freeze dryer (Shanghai Tianfeng Industrial Co., Ltd.), refrigerated ultracentrifuge (Thermo Corporation, USA), and an ultrasound system (General Electric Co., USA; model GE LOGIQ E11) were used in this study.

### 2.4 Construction of the LMS–disease Target Network Diagram

The active ingredients of each herb in LMS were obtained from TCMSP (http://tcmspw.com/tcmsp.php) and ETCM (http://www.tcmip.cn/ETCM/). The active ingredients were screened using oral bioavailability (OB) ≥ 30% and drug-likeness (DL) ≥ 0.18 as the selection criteria, and the potential targets of these ingredients were identified.

The thresholds for OB (≥ 30%) and DL (≥ 0.18) were chosen based on well-established pharmacological principles and prior literature. OB reflects the proportion of a drug that reaches systemic circulation after oral administration and is an important indicator of absorption efficiency. Studies have shown that many clinically effective oral drugs exhibit OB values above 30%, whereas compounds with OB < 30% often fail to achieve adequate pharmacological activity *in vivo* [38]. DL evaluates the structural and physicochemical similarity of compounds to known drugs. A DL value greater than 0.18 typically suggests that a compound possesses properties and pharmacokinetic characteristics similar to existing drugs and may have drug-like potential [38]. Therefore, applying OB ≥ 30% and DL ≥ 0.18 as screening thresholds helps identify components likely to exert practical biological activity *in vivo*, and these critical values have been widely adopted in many studies [39].

The GEO database was searched using the keyword “lipid metabolism disorders” to identify relevant disease models. The GSE133400 dataset was selected to determine disease-related target genes. Cytoscape (version 3.7.1) was then used to construct an interaction network between the active ingredients of each LMS herb and the disease targets.

### 2.5 Construction of a Protein–protein Interaction (PPI) Network Diagram

In the GEO database, the keyword “lipid metabolism disorder” was used to retrieve relevant disease models, and the GSE133400 dataset was selected to identify disease-related target genes. By intersecting the targets associated with lipid metabolism disorders and those related to LMS, potential therapeutic targets of LMS were identified.

The overlapping targets were imported into the STRING database to generate the PPI network. To ensure data reliability, a minimum combined interaction score threshold of 0.400 was applied, and the analysis was restricted to *Homo sapiens*. Cytoscape (version 3.7.3) was then used to construct and analyze the PPI network of the potential therapeutic targets of the active metabolites in LMS.

### 2.6 GO and KEGG Enrichment Analyses

Gene Ontology (GO) and Kyoto Encyclopedia of Genes and Genomes (KEGG) enrichment analyses were performed on the basis of the targets of LMS. GO enrichment analysis revealed the physiological functions and cellular components associated with the therapeutic targets [in the biological process (BP), molecular function (MF), and cellular component (CC) categories]. KEGG enrichment analysis revealed the metabolic pathways associated with the therapeutic targets and identified the metabolic pathways and physiological processes that affect the development of lipid metabolism disorders.

### 2.7 Cell Model

To establish a cell model of high-fat conditions, HepG2 cells were treated with 1 mM oleic acid for 24 h to induce lipid droplet formation. The cells in the blank control group were not treated with oleic acid. After HepG2 cells were treated with 0 μg, 10 μg, or 30 μg of LMS for 24 h, the high- fat model cells were subjected to immunofluorescence analysis or lysed to obtain cell extracts for subsequent biochemical analyses.

### 2.8 Measurement of Cellular Biochemical Indicators

Kits provided by Nanjing Jiancheng Bioengineering Institute were used to measure total cholesterol (TC) and triglyceride (TG) in cell lysates, and the lysate protein content was used as a measure of cell number to normalize the data.

### 2.9 Immunofluorescence Analysis of Cells

Cells treated as described above were cultured on glass slides for 24 h, fixed with 4% PFA at room temperature for 30 min, washed twice with PBS, and blocked with 10% goat serum. The cells were incubated with primary antibodies specific for SREBP1 and PPARγ (1:500, Proteintech) at 4°C overnight. After the cells were washed, they were incubated with a FITC-conjugated secondary antibody (1:1000, Proteintech) at room temperature for 1 h in the dark. The solution containing the secondary antibody was removed, and the cells were incubated in DAPI staining solution for 45 min and then washed. The cells were mounted with an anti-fluorescence quenching reagent and observed under a fluorescence microscope. ImageJ was used to calculate the regional fluorescence intensity. Polyclonal antibodies specific for SREBP1 and PPARγ and goat anti-mouse IgG (H+L) were purchased from Proteintech.

### 2.10 Animal Experiment

After adaptive feeding for 1 week, SD rats were randomly divided into 3 groups, with 6 rats in each group: the normal control (CON) group, the high-fat diet (HFD) model group, and the LMS treatment group. The rats in the CON group were fed normal chow during the experiment. The rats in the HFD model group and the LMS group were fed an HFD. The rats in the LMS group were also gavaged with LMS solution (200 mg/(kg·d)) for 12 weeks. Body weight and food intake were recorded once a week.

### 2.11 Ultrasound Examination

The rat liver was examined by ultrasound for dynamic detection of the progression of lipid metabolism disorders. The maximum diameter of the right liver lobe was measured with a probe (Model L8-18i) at the oblique section of the subcostal liver border where the right hepatic vein joins the inferior vena cava. The thickness and length of the left liver lobe were measured on sagittal longitudinal sections that included the abdominal aorta. The liver volume and echo intensity were recorded.

### 2.12 Animal Sample Collection

Before the end of the experiment, the rats were fasted for 12 h with free access to water. Mice were anesthetized with isoflurane to minimize pain and subsequently euthanized with carbon dioxide. Blood was collected from the orbital venous plexus at the time of sacrifice and placed in heparinized centrifuge tubes. Plasma was isolated by centrifugation at 3000 × g for 10 minutes at 4°C and stored at -80°C for biochemical analysis. After sacrifice, liver tissue was rapidly excised, rinsed with 0.9% sodium chloride solution, and fixed in 4% paraformaldehyde. The remaining liver tissue was stored at -80°C for further analyses.

### 2.13 Measurement of Biochemical Indicators

The TC, TG, low-density lipoprotein cholesterol (LDLC), high-density lipoprotein cholesterol (HDLC), aspartate aminotransferase (AST), and alanine aminotransferase (ALT) concentrations in plasma were measured with kits provided by Nanjing Jiancheng Bioengineering Institute. The levels of TC and TG in the liver were measured with kits provided by Nanjing Jiancheng Bioengineering Institute. The levels of IL-6 and TNF-α in the liver were measured with kits provided by Cusabio Bioengineering Co., Ltd.

### 2.14 Pathological Evaluation of the Liver

Fixed liver tissue was collected and dehydrated through an ethanol gradient until transparent. Tissues were embedded in paraffin, sectioned, stained with hematoxylin and eosin (H&E), and mounted. Pathomorphological changes in liver tissues were evaluated by microscopy (40×).

### 2.15 Immunofluorescence Analysis

Paraffin sections of liver tissue were obtained, dewaxed in xylene, hydrated through an ethanol gradient, subjected to antigen retrieval, and blocked with 10% goat serum. The sections were incubated with primary antibodies specific for SREBP1 and PPARγ (1:500, Proteintech) at 4°C overnight. After the sections were washed overnight, they were incubated with a FITC-conjugated secondary antibody (1:1000, Proteintech) at room temperature for 1 h in the dark. The secondary antibody solution was removed, and the sections were incubated in DAPI staining solution for 45 min and then washed. The sections were mounted with an anti-fluorescence quenching reagent and observed under a fluorescence microscope. ImageJ was used to calculate the regional fluorescence intensity.

### 2.16 Statistical Methods

Data analysis and graphing were performed with GraphPad Prism 8. A t-test was used to determine the significance of differences between two groups, and ANOVA followed by Dunnett's post hoc test was used for comparisons among multiple groups. A *P* value <0.05 indicated a significant difference, and a *P* value <0.01 indicated a highly significant difference.

## 3 RESULTS

### 3.1 Construction of the Active Ingredient‒target Network

After screening the TCMSP and ETCM databases, a total of 32 active ingredients and 263 potential targets were screened (Fig. **1a**). Active ingredient–target networks were constructed *via* Cytoscape 3.7.2. The active ingredients and targets of each herb are shown in Table **2** (details are given in Table **SI**)

### 3.2 PPI Network Diagram

On the basis of the GSE133400 dataset in the GEO database, 499 disease genes were identified *via* analysis of the raw sequencing data for rats in the CON and HFD groups (N=4 rats/group) (Table **SII**). The action targets of LMS and the disease genes were used for Venn diagram analysis, and the intersection was obtained. A total of 29 overlapping target genes, including PPARG and SREBF1, were identified. The 29 overlapping target genes were imported into STRING and Cytoscape, and 11 overlapping target genes were selected to construct a PPI network diagram (Fig. **1b**). The core target genes and related information are listed in Table **3**. CytoHubba was used to analyze the key nodes of the PPI network, and SREBF1 and PPARG were identified as potential therapeutic targets of LMS (Fig. **1c**).

### 3.3 GO and KEGG Enrichment Analyses

GO and KEGG enrichment analyses were performed with the human genome as the reference. Regarding therapeutic targets, as shown in Fig. (**2a**), the most significantly enriched biological processes were the regulation of lipid metabolism and cholesterol metabolism. As shown in Fig. (**2b**), the most significantly enriched cellular components were HDL particle and plasma lipoprotein particle. As shown in Fig. (**2c**), the most significantly enriched molecular function was transcription factor activity. As shown in Fig. (**2d**), the most significantly enriched signaling pathway related to lipid metabolism was the peroxisome proliferator-activated receptor (PPAR) signaling pathway.

### 3.4 Effects of LMS on Lipid Accumulation in HepG2 Cells

Compared to the control treatment, oleic acid significantly increased the Oil Red O content in HepG2 cells and induced fat accumulation in HepG2 cells, whereas LMS significantly reduced the Oil Red O content and fat accumulation in HepG2 cells (Fig. **3a**). Measurement of the intracellular TG and TC contents (Figs. **3b** and **c**) revealed that in HepG2 cells, oleic acid significantly increased the TC and TG contents, which decreased significantly after treatment with LMS (*P* <0.01).

### 3.5 Effects of LMS on SREBP1 and PPARγ Expression in HepG2 Cells

The immunofluorescence results are shown in Figs. (**4a** and **b**). Compared with the control treatment, oleic acid significantly increased the nuclear expression level of SREBP1 in HepG2 cells and decreased the cytoplasmic expression level of SREBP1. After HepG2 cells were treated with LMS, the nuclear SREBP1 expression level decreased significantly, and the cytoplasmic SREBP1 expression level increased significantly. Furthermore, after HepG2 cells were treated with LMS, the nuclear expression level of PPARγ increased significantly, and the cytoplasmic expression level of PPARγ decreased significantly. These results suggest that LMS can inhibit SREBP1 nuclear transcription and activate PPARγ nuclear transcription.

### 3.6 Effects of LMS on the Body Weight and Food Intake of HFD-fed Rats

Fig. (**5a**) shows a flow chart of the animal experiments. As shown in Figs. (**5b** and **5c**), compared with the rats in the CON group, the rats in the HFD group had significantly increased body weights (*P* <0.001) and significantly reduced food intake (*P* <0.05). However, there were no significant differences in body weight or food intake between the LMS group and the HFD group.

### 3.7 Effects of LMS on the Livers of HFD-fed Rats by Dynamic Ultrasound

Fig. (**5d**) shows that at weeks 4 and 12, in the CON group, the liver volume was normal, there was a slight increase in echogenicity, the signals were sharp and evenly distributed, and far-field echo attenuation was not obvious. However, in the HFD group, liver volume was increased, echogenicity was significantly increased, and signals were sharp; the intrahepatic duct structure was indistinct, and the far-field signal was significantly attenuated. The liver volume was significantly lower, the liver echoes were significantly attenuated and sharper, the relative distribution was more uniform, and the far-field signals were slightly weaker in the LMS group compared with the HFD group.

### 3.8 Effects of LMS on Plasma Physiological and Biochemical Indicators in HFD-fed Rats

The plasma TC (*P* < 0.05), TG (*P* < 0.01), LDLC (*P* < 0.05), and HDLC (*P* < 0.01) levels were significantly greater in the HFD group than in the CON group (Figs. **6a**-**d**). The plasma TC (*P* < 0.01), TG (*P* < 0.05), and LDLC (*P* < 0.05) levels were significantly lower in the LMS treatment group than in the HFD group, but the HDLC level did not differ significantly between these groups Fig. (**6**). As shown in Figs. (**6g** and **h**), the AST and ALT levels in rat plasma were significantly greater in the HFD group than in the CON group (*P* <0.01); in addition, the AST (*P* < 0.01) and ALT (*P* < 0.01) levels in rat plasma were significantly lower in the LMS group than in the HFD group. These results suggest that LMS significantly alleviated lipid metabolic disorders and abnormal liver function induced by HFD feeding.

### 3.9 Effects of LMS on Liver Physiological and Biochemical Indicators in HFD-fed Rats

Compared with those in the livers of the rats in the CON group, the TC (*P* < 0.01) and TG (*P* < 0.01) levels in the livers of the rats in the HFD group were significantly increased (Figs. **6e** and **f**); compared with those in the livers of the rats in the HFD group, the TC and TG levels in the livers of the rats in the LMS group were significantly decreased (*P* <0.01). As shown in Figs. (**6i** and **j**), compared with those in the livers of the rats in the CON group, the levels of TNF-α (*P* < 0.01) and IL-6 (*P* < 0.01) in the livers of the rats in the HFD group were significantly increased; compared with those in the livers of the rats in the HFD group, the levels of TNF-α (*P* < 0.05) and IL-6 (*P* < 0.05) in the livers of the rats in the LMS group were significantly decreased.

### 3.10 Effect of LMS on the Pathological Histomorphology of the Liver in HFD-fed Rats

Significant morphological differences were observed in the cells in the liver tissue between the rats in the HFD group, which exhibited shrunken nuclei, significantly more intracellular fat vacuoles, narrower intercellular gaps, and tighter cell adhesions, and the rats in the CON group (Fig. **6k**). However, compared with the corresponding indicators in rats in the HFD group, the cell morphology of liver tissue from rats in the LMS group showed significant improvement. The degree of hepatocyte nuclear shrinkage was notably reduced, while the number and size of cytoplasmic fat vacuoles decreased significantly, accompanied by widened intercellular spaces. Nevertheless, the morphological features of the liver tissue had not yet fully returned to normal control.

### 3.11 Effects of LMS on SREBP1 and PPARγ Expression in the Liver in HFD-fed Rats

Immunofluorescence staining (Fig. **7a**) revealed that, compared with that in the liver cells of the rats in the CON group, the nuclear SREBP1 expression level in the liver cells of the rats in the HFD group was significantly increased, whereas LMS treatment decreased the nuclear SREBP1 expression level in the liver cells of the rats fed the HFD. Moreover, as shown in Fig. (**7b**), LMS treatment increased the nuclear expression level of PPARγ in the liver cells of rats fed the HFD. These results suggest that LMS can inhibit SREBP1 transcriptional activity and activate PPARγ transcriptional activity.

## 4 DISCUSSION

Lipid metabolism disorders mainly include blood lipid metabolism disorders and liver lipid metabolism disorders; therefore, multitarget treatment is essential [40]. Although the clinical efficacy of LMS in treating lipid metabolism disorders has been established, its underlying mechanisms remain unclear. Through ultrasound examination, histopathological analysis, and other techniques, we demonstrated that LMS significantly reduced lipid accumulation in liver cells and improved plasma lipid profiles both *in vivo* and *in vitro*. Network analysis and immunofluorescence staining further confirmed that SREBP1 and PPARγ were the main therapeutic regulatory targets. LMS inhibits SREBP1 transcriptional activity and activates PPARγ transcriptional activity to alleviate lipid metabolism disorders.

SREBP1 is a key regulator of cholesterol anabolism [41-43]. FASN and ACC are critical rate-limiting enzymes involved in fatty acid synthesis [44], and SREBP1 regulates their expression [45, 46]. Once activated, SREBP1 translocates into the nucleus, binds to the promoters of key genes involved in fatty acid synthesis, increases fatty acid production, and elevates TG levels in the liver and serum [47]. Our *in vivo* and *in vitro* results showed that LMS significantly inhibited SREBP1 transcriptional activity, which represents one of its primary lipid-lowering mechanisms. Kaempferol, derived from *C. pinnatifida* and *H. dulcis*, effectively inhibits SREBP1 expression and reduces cholesterol synthesis [48]. In addition, STAT3 is an important transcriptional activator that regulates SREBP1 and other transcription factors [49]. Quercetin derived from *P. sinensis*, *C. pinnatifida*, and *H. dulcis* can inhibit STAT3 phosphorylation and the expression of SREBP1 and related cholesterol synthases, thereby reducing hepatic lipid accumulation [50-53]. Furthermore, genistein from *M. purpureus* can regulate AMPK activity, affect SREBP1 expression, reduce HMGCR levels, and inhibit cholesterol synthesis [54, 55]. Therefore, active metabolites from *C. pinnatifida*, *H. dulcis*, *M. purpureus*, and *P. sinensis* may underlie the LMS-mediated reductions in liver and serum TG levels through the targeting of SREBP1.

PPARs are nuclear receptors involved in physiological processes such as lipid metabolism, cell proliferation, and cell differentiation [56]. PPARs are classified into three subtypes: α, β/δ, and γ. PPARγ, encoded by *PPARG*, is expressed in liver tissue and participates in processes including cholesterol metabolism and inflammation [57]. PPARγ activation promotes cholesterol efflux and reduces inflammation. Once activated, PPARγ can further stimulate LXRα, upregulate ABCA1 expression, promote cholesterol efflux, and decrease hepatic TC levels [58]. In addition, PPARγ plays an important role in regulating insulin resistance in mice, increasing insulin sensitivity, and exerting positive therapeutic effects on NAFLD [59, 60]. Our *in vivo* and *in vitro* results revealed that LMS significantly activated PPARγ transcriptional activity, representing another major mechanism of its lipid-lowering action. The active metabolites of *P. sinensis*, *C. pinnatifida*, and *H. dulcis* also modulate PPAR activity and reduce lipid accumulation in mice. These three medicinal herbs may therefore form the basis of the LMS-induced reductions in liver and serum TC levels through the targeting of PPARγ.

Studies have shown that herbs such as *G. lucidum* and *P. cocos*, which are components of LMS, can reduce lipid accumulation in the liver. Stigmasterol and β-sitosterol derived from *G. lucidum* can ameliorate HFD-induced fatty liver, as indicated by reductions in total lipid content, cholesterol content in the liver, and the severity of liver tissue lesions [61, 62]. The mechanism may involve decreasing bile acid levels in the intestines and inhibiting intestinal fat absorption. Hederagenin derived from *P. cocos* can regulate the synthesis and oxidation of lipids and cholesterol; however, its underlying mechanism remains unclear and requires further study [63]. In addition, the therapeutic efficacy of Linggui Zhugan Decoction—which contains the traditional Chinese medicinal herbs *P. cocos* and *G. lucidum*—in lipid metabolism disorders has been verified in mouse experiments [64, 65].

Lipid metabolism disorders induce inflammation, and inflammation further exacerbates lipid metabolism disorders, jointly contributing to the development of metabolic diseases [66]. Dysregulated inflammatory cytokine expression in the liver caused by lipid metabolism disorders is often associated with persistent activation of the JAK–STAT signaling pathway [67]. Quercetin can significantly reduce hepatic IL-1β levels and alleviate liver inflammation by regulating the Farnesoid X receptor 1/Takeda G-protein-coupled receptor 5 signaling pathway [68-73]. In addition, compounds found in *P. sinensis*, *C. pinnatifida*, and *M. purpureus* can inhibit liver inflammation, thereby slowing disease progression associated with lipid metabolism disorders [74, 75].

## 5 STUDY LIMITATIONS

Several limitations of this study should be acknowledged. First, while the high-fat diet-induced rat model and oleic acid-stimulated HepG2 cells are widely used for studying lipid metabolism disorders, they may not fully recapitulate the complexity of human metabolic diseases. Second, the sample size in animal experiments was relatively small, and the treatment duration was limited to 12 weeks; longer-term studies are required to assess the sustainability and potential adverse effects of LMS. Third, the present study focused primarily on SREBP1 and PPARγ pathways; other possible mechanisms, such as inflammation, oxidative stress, and gut microbiota modulation, were not comprehensively investigated. Finally, the absence of clinical data restricts the translational relevance of our findings. Future clinical trials are warranted to evaluate the efficacy and safety of LMS in patients with lipid metabolism disorders. Despite these limitations, our study provides a solid foundation for understanding the therapeutic mechanisms of LMS and offers a methodological framework for investigating complex traditional Chinese medicine formulations.

## CONCLUSION

In summary, the active ingredients of LMS may regulate processes such as cholesterol anabolism and lipid anabolism by modulating key targets, including SREBP1 and PPARγ, thereby reducing lipid accumulation in the liver. This may represent an important regulatory mechanism by which LMS exerts therapeutic effects on lipid metabolism disorders. Although this study provides preliminary confirmation of the relationships among LMS, SREBP1, and PPARγ, further research is needed to verify the interactions between the active ingredients and their therapeutic targets.

In this study, we not only demonstrated that SREBP1 and PPARγ are important targets of LMS in treating lipid metabolism disorders but also applied predictive network analysis combined with biological experimental validation to offer a framework for future investigations into the pharmacological mechanisms of other metabolites in TCMs used for the treatment of lipid metabolism disorders and related chronic metabolic diseases.

## Figures and Tables

**Fig. (1):**
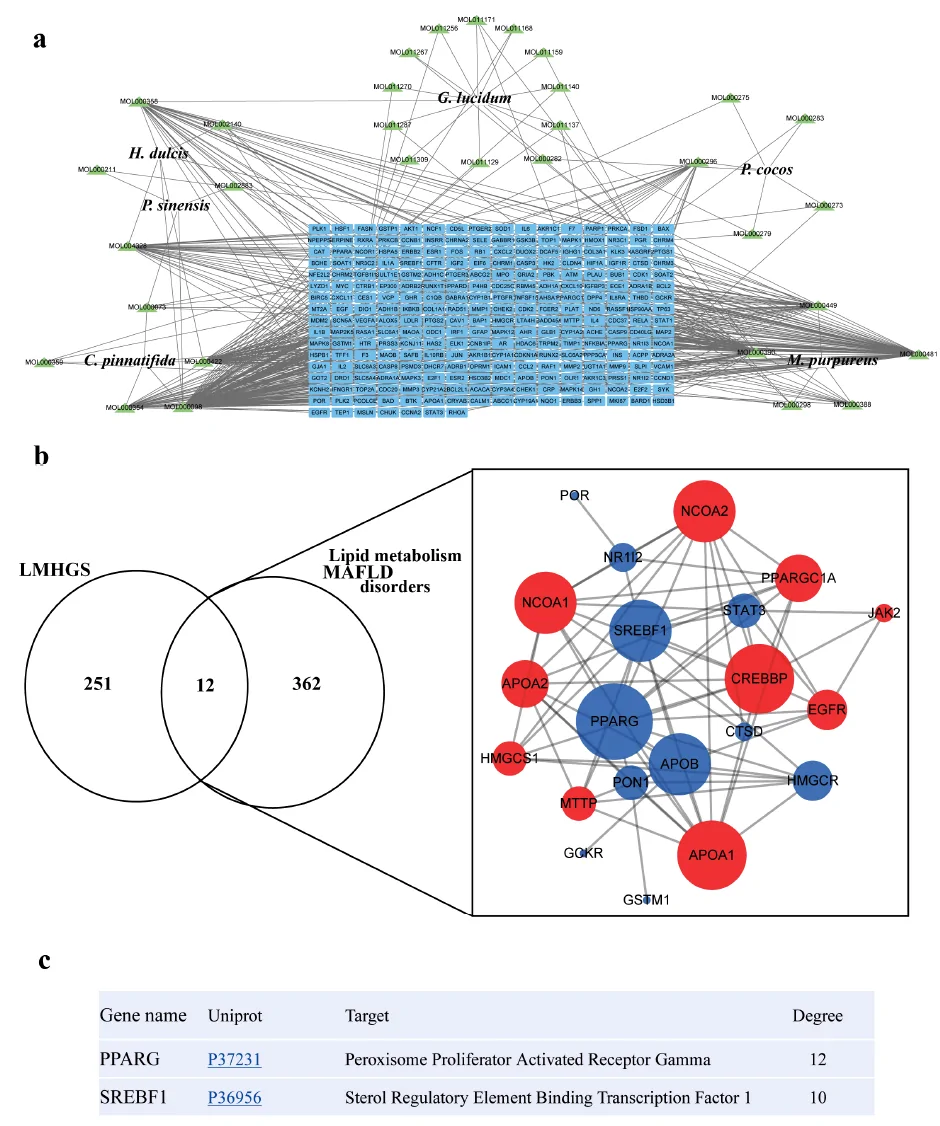
Therapeutic targets of LMS for lipid metabolism disorders and the bioactive ingredient–target network. (**a**) Interaction network between the active ingredients of each herb in LMS and the disease genes. (**b**) Venn diagram showing the overlap between the potential LMS target genes and NAFLD-related genes and the PPI network of the overlapping genes. (**c**) The potential therapeutic targets of LMS are PPARG and SREBF1.

**Fig. (2):**
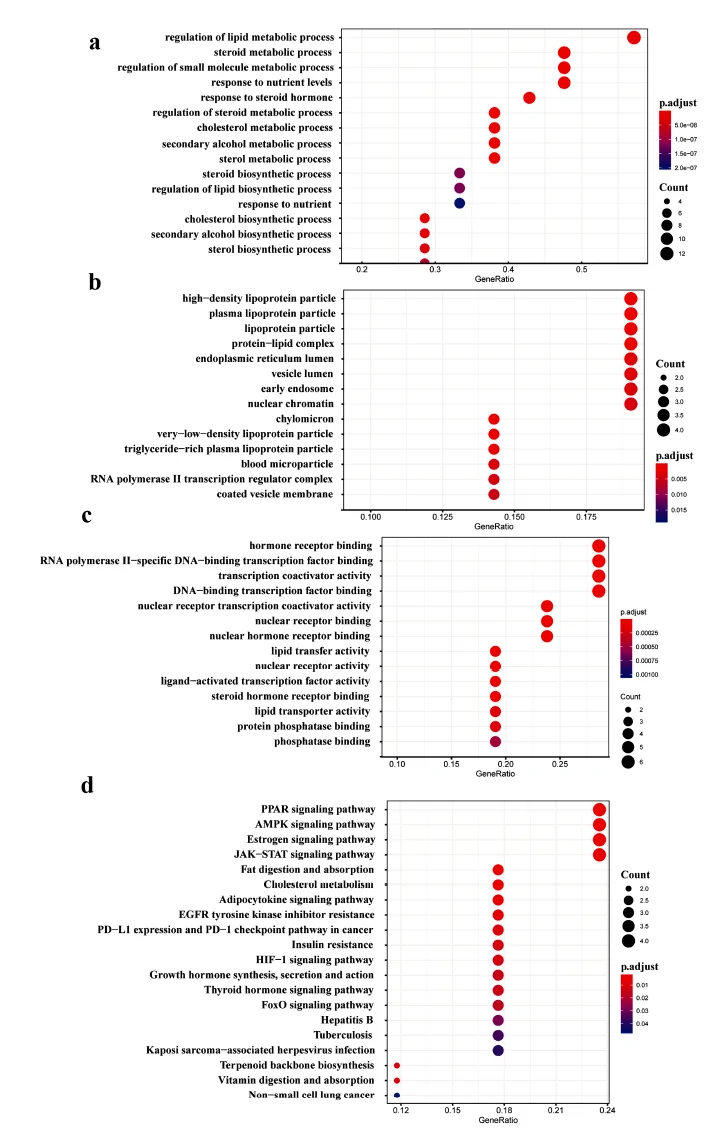
GO and KEGG enrichment analysis results. Results of GO term enrichment analysis of the potential target genes of LMS: (**a**) biological processes, (**b**) cellular components, (**c**) molecular functions. (**d**) Results of KEGG pathway enrichment analysis.

**Fig. (3):**
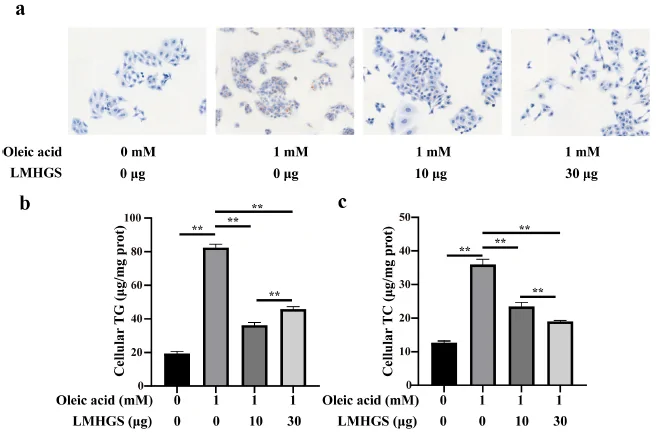
Effect of LMS on lipid accumulation in HepG2 cells induced by oleic acid. (**a**) Lipids in HepG2 cells were detected by Oil Red O staining. (**b**) Cellular TG concentration. (**c**) Cellular TC concentration. The data are presented as the means ± SEMs (n =3 experimental replicates). Significant differences were identified (** *P*<0.01) *via* one-way ANOVA with Dunnett's post hoc test.

**Fig. (4):**
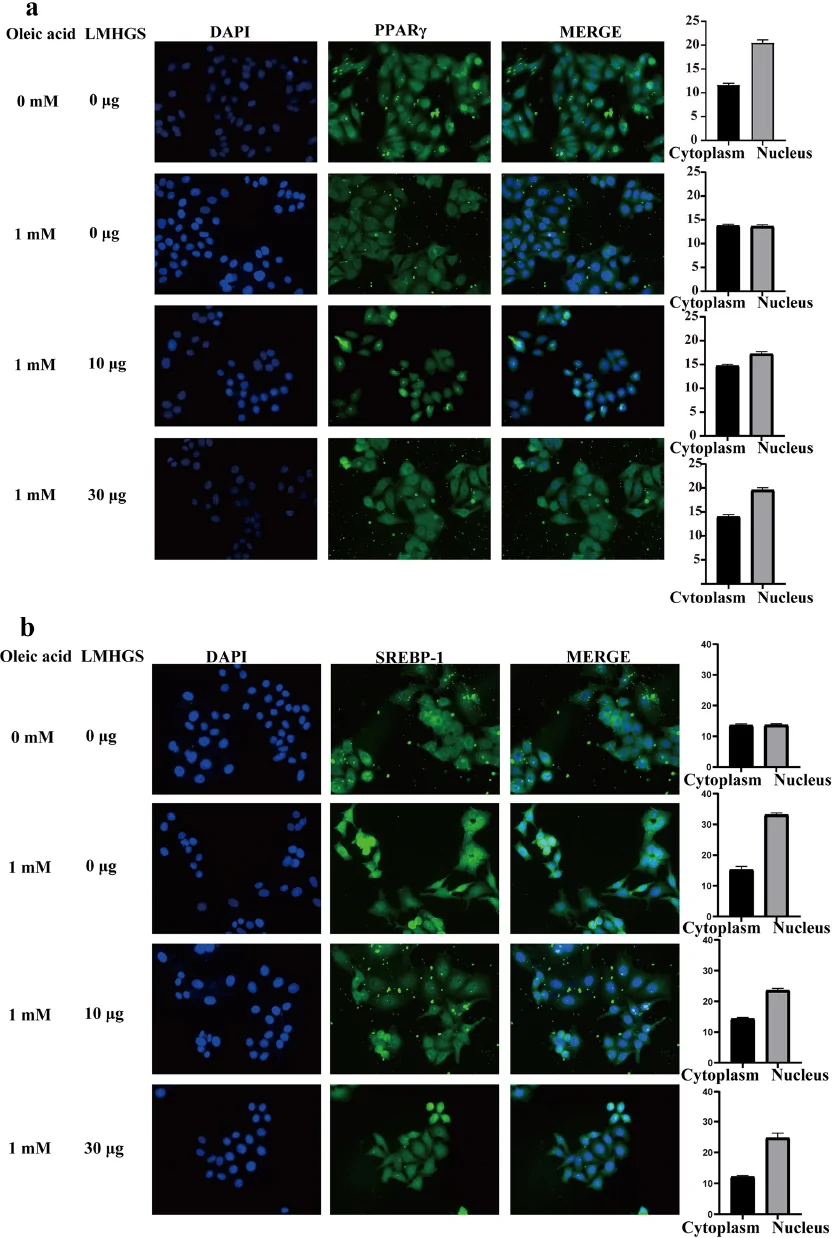
Effect of LMS on the expression of SREBP1 and PPARγ in HepG2 cells induced by oleic acid. (**a, b**) The expression of PPARγ and SREBP1 in HepG2 cells was evaluated by immunofluorescence staining. The histogram shows the expression levels of PPARγ and SREBP1 in the cytoplasm and nucleus.

**Fig. (5):**
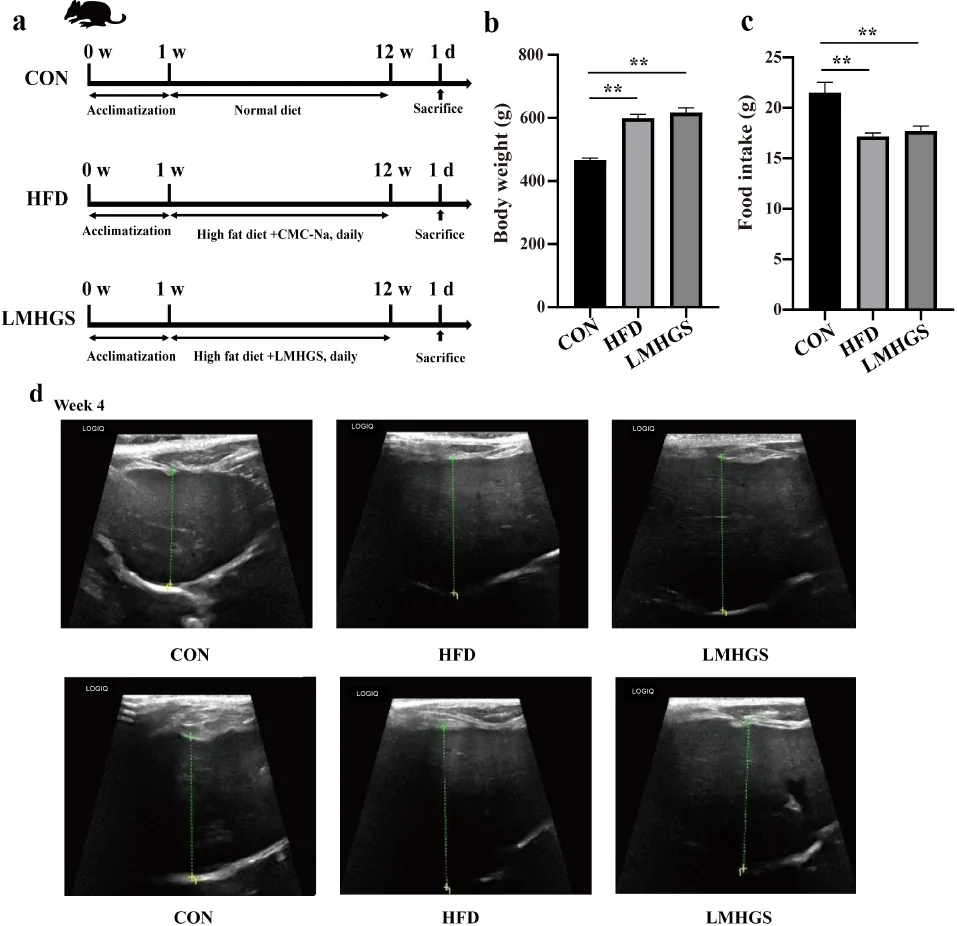
Effects of LMS on body weight, food intake and liver ultrasound findings in HFD-fed rats. (**a**) Design and timeline of the animal experiments. (**b**) Body weight. (**c**) Food intake. (**d**) Ultrasound images of the livers of rats at weeks 4 and 12. The data are presented as the means ± SEMs (n = 6 rats/group). Significant differences were identified (** *P*<0.01) *via* one-way ANOVA with Dunnett's post hoc test.

**Fig. (6):**
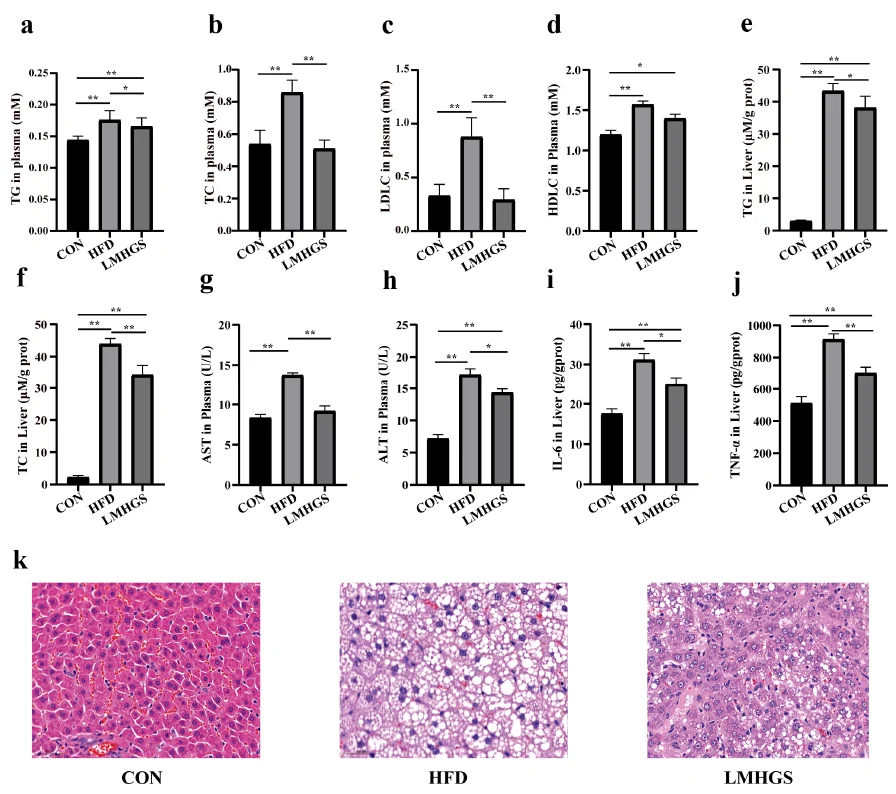
Effects of LMS on plasma and liver physiological and biochemical indices in HFD-fed rats. (**a**) Plasma TG; (**b**) plasma TC; (**c**) plasma LDLC; (**d**) plasma HDLC; (**e**) hepatic TG; (**f**) hepatic TC; (**g**) plasma AST; (**h**) plasma ALT; (**i**) hepatic IL-6; (**j**) hepatic TNF-α. (**k**) HE staining of liver tissue from rats. The data are presented as the means ± SEMs (n = 6 rats/group). Significant differences were identified (*P<0.05 and **P<0.01) *via* one-way ANOVA with Dunnett's post hoc test.

**Fig. (7):**
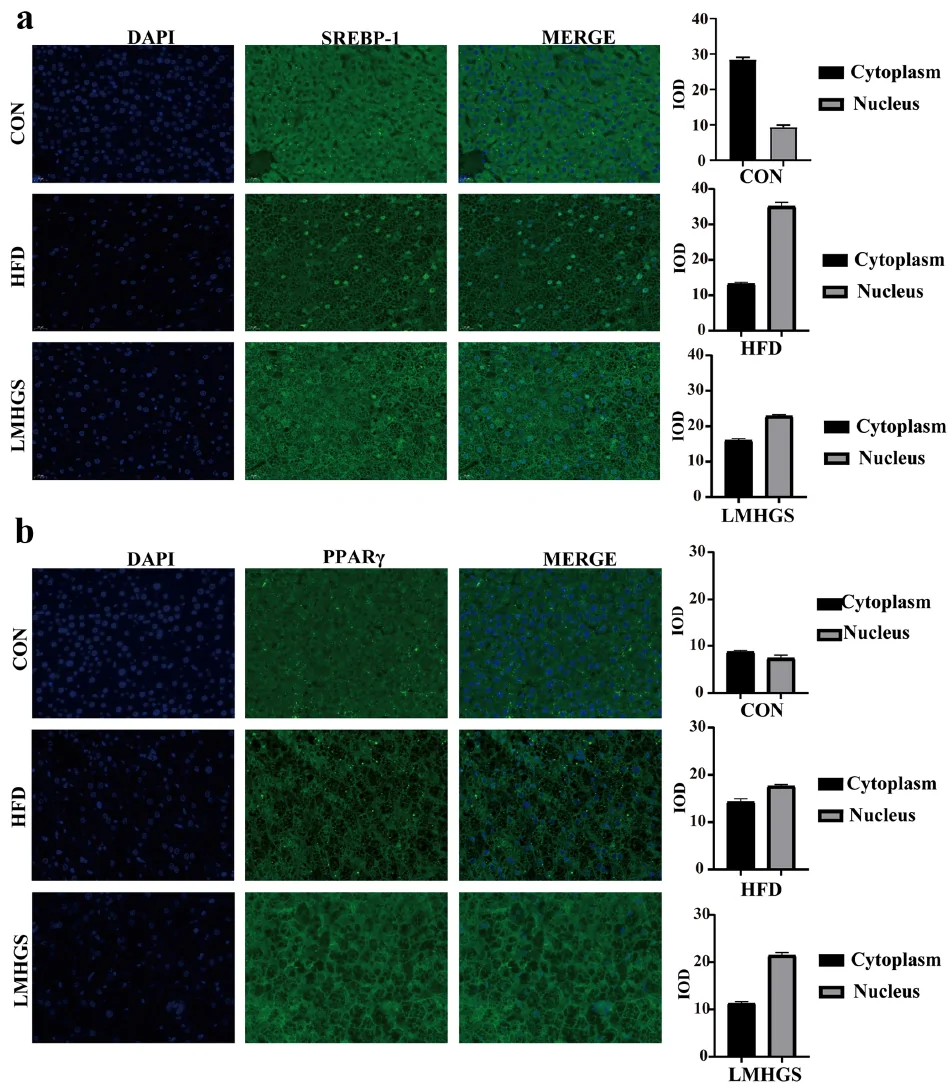
Effects of LMS on SREBP1 and PPARγ expression in HFD-fed rats. (**a**) Immunofluorescence staining for SREBP1 in liver tissue (original magnification, 200×); the histogram shows SREBP1 expression in the cytoplasm and nucleus. (**b**) Immunofluorescence staining for PPARγ in liver tissue (original magnification, 200×); the histogram shows SREBP1 expression in the cytoplasm and nucleus.

**Table 1:** Detailed composition of the diets.

-	Normal Chow-group	-	High-Fat Diet (HFD)-group	-
Ingredient	gm	kcal	gm	kcal
Casein, 80 MMfesh	200	800	200	800
L-Cystine	3	12	3	12
Corn·Starch	397	1590	0	0
Maltodextrin 10	132	528	125	500
Sucrose	100	400	68.8	275.2
Cellulose. ·BW200	50	0	50	0
Soybean·Oil	70	630	25	225
t-Butylhydroquinone	0.014	0	0	0
Mineral·Mix·S10022G	35	0	0	0
Vitamin·Mix·V10037	10	40	0	0
Lard	0	0	245	2205
Mineral·Mix, ·S10026	0	0	10	0
DiCalcium·Phosphate	0	0	13	0
Calcium·Carbonate	0	0	5.5	0
Potassium·Citrate. 1·H20	0	0	16.5	0
Vitamin·Mix, V10001	0	0	10	40
Choline·Bitartrate	2.5	0	2	0
FD&C·Blue·Dye	0	0	0.05	0
Polyene·phosphatidyl·choline	0	0	0	0
Total	999.514	4000	773.95	4057.2

**Table 2:** Effective ingredients and targets of LMS.

TCM	Number of Effective Ingredients	Number of Targets
*P. cocos*	6	18
*M. purpureus*	5	152
*G. lucidum*	14	30
*P. sinensis*	4	139
*C. pinnatifida*	6	169
*H. dulcis*	6	184

**Note:** The ingredients and targets were obtained from the ETCM database.

**Table 3:** Core regulatory genes and related information.

Gene Name	UniProt ID	Target	Degree
PPARG	P37231	Peroxisome Proliferator Activated Receptor Gamma	12
SREBF1	P36956	Sterol Regulatory Element Binding Transcription Factor 1	10
HMGCR	P00347	3-hydroxy-3-methylglutaryl-coenzyme A reductase	7
STAT3	P40763	Signal transducer and activator of transcription 3	6

## Data Availability

The data that support the findings of this study are available from the corresponding author, [F.-J.B.] upon special request.

## LIST OF ABBREVIATIONS

ALT: Alanine Aminotransferase
ARRIVE: Animal Research: Reporting of *In vivo* Experiments
AST: Aspartate Aminotransferase
DAPI: 4',6-diamidino-2-phenylindole
DL: Drug Likeness
ETCM: Ethnomedicinal Chinese Medicine Database
GEO: Gene Expression Omnibus
GO: Gene Ontology
H&E: Hematoxylin and Eosin
HFD: High-fat Diet
HDL-C: High-density Lipoprotein Cholesterol
IL-6: Interleukin-6
KEGG: Kyoto Encyclopedia of Genes and Genomes
LDL-C: Low-density Lipoprotein Cholesterol
OB: Oral Bioavailability
PPAR: Peroxisome Proliferator-activated Receptor
PPARγ: Peroxisome Proliferator-activated Receptor Gamma
SD: Sprague Dawley
SREBP: Sterol Regulatory Element Binding Protein
SREBP1: Sterol Regulatory Element Binding Transcription Factor 1
TC: Total Cholesterol
TCMSP: Traditional Chinese Medicine Systems Pharmacology Database
TG: Triglyceride
TNF-α: Tumor Necrosis Factor-alpha
