# Supplementary material for: Investigation of the Mechanism of Action of Liu Miao San in the Treatment of Lipid Metabolism Disorders on the basis of Network Analysis and Dynamic Ultrasound
Source: Endocr Metab Immune Disord Drug Targets. 2026 May 21;26:E18715303417569. doi: 10.2174/0118715303417569251201075052 (PMC13670187; doi:10.2174/0118715303417569251201075052)
Supplement: Supplementary file 1 [file EMIDDT-26-E18715303417569_SD1.pdf]

Supplementary Material

Investigation of the Mechanism of Action of *Liu Miao San* in the Treatment of Lipid Metabolism Disorders on the basis of Network Analysis and Dynamic Ultrasound

Yu-Jia Xu[1], Hua Hou[2], Lin Liu[2] and Fang-Jie Bi[2,*]

[1]Department of Echocardiography, Zibo Central Hospital, No. 54 Zhangdian District - Communist Youth League West Road, Zibo, 255000, Shandong Province, People's Republic of China; [2]Department of Heart Center, Zibo Central Hospital, No. 54 Zhangdian District - Communist Youth League West Road, Zibo, 255000, Shandong Province, People's Republic of China

Table SI. Corresponding information of effective ingredients and targets.

TCM	MolId	MolName	Symbol
P.cocos	MOL000273	(2R)-2-[(3S,5R,10S,13R,14R,16R,17R)-3,16-dihydroxy-4,4,10,13,14-pentamethyl-2,3,5,6,12,15,16,17-octahydro-1H-cyclopenta[a]phenanthren-17-yl]-6-methylhept-5-enoic acid	NR3C2
P.cocos	MOL000273	(2R)-2-[(3S,5R,10S,13R,14R,16R,17R)-3,16-dihydroxy-4,4,10,13,14-pentamethyl-2,3,5,6,12,15,16,17-octahydro-1H-cyclopenta[a]phenanthren-17-yl]-6-methylhept-5-enoic acid	NCOA2
P.cocos	MOL000275	trametenolic acid	NR3C2
P.cocos	MOL000279	Cerevisterol	NR3C2
P.cocos	MOL000282	ergosta-7,22E-dien-3beta-ol	PGR
P.cocos	MOL000283	Ergosterol peroxide	PGR
P.cocos	MOL000296	hederagenin	PGR
P.cocos	MOL000296	hederagenin	NCOA2
P.cocos	MOL000296	hederagenin	CHRM3
P.cocos	MOL000296	hederagenin	CHRM1
P.cocos	MOL000296	hederagenin	CHRM2
P.cocos	MOL000296	hederagenin	ADRA1B
P.cocos	MOL000296	hederagenin	GABRA1

P.cocos	MOL000296	hederagenin	GRIA2
P.cocos	MOL000296	hederagenin	IGHG1
P.cocos	MOL000296	hederagenin	ADH1B
P.cocos	MOL000296	hederagenin	ADH1C
P.cocos	MOL000296	hederagenin	LYZD1
P.cocos	MOL000296	hederagenin	PTGS1
P.cocos	MOL000296	hederagenin	SCN5A
P.cocos	MOL000296	hederagenin	PTGS2
P.cocos	MOL000296	hederagenin	RXRA
P.cocos	MOL000296	hederagenin	SLC6A2
G.lucidum	MOL011129	methyl (4R)-4-[(5R,10S,13R,14R,17R)-4,4,10,13,14-pentamethyl-3,7,11,15-tetraoxo-2,5,6,12,16,17-hexahydro-1H-cyclopenta[a]phenanthren-17-yl]pentanoate	NR3C2
G.lucidum	MOL011137	campesta-7,22E-dien-3beta-ol	PGR
G.lucidum	MOL011137	campesta-7,22E-dien-3beta-ol	NR3C2
G.lucidum	MOL011137	campesta-7,22E-dien-3beta-ol	NCOA2
G.lucidum	MOL011140	5alpha-Lanosta-7,9(11),24-triene-15alpha,26-dihydroxy-3-one	NR3C2
G.lucidum	MOL011159	ergosta-4,6,8(14),22-tetraene-3-one	PGR
G.lucidum	MOL011168	ergosta-7,9(11),22-trien-3尾,5伪,6伪-triol	NR3C2
G.lucidum	MOL011171	ganoderal B	PGR
G.lucidum	MOL011171	ganoderal B	NCOA2
G.lucidum	MOL011256	ganolucidic acid E	NR3C2
G.lucidum	MOL011267	Lucialdehyde B	PGR
G.lucidum	MOL011270	(4R)-4-[(5R,7S,10S,13R,14R,17R)-7-hydroxy-3,11,15-triketo-4,4,10,13,14-pentamethyl-1,2,5,6,7,12,16,17-octahydrocyclopenta[a]phenanthren-17-yl]valeric acid	NR3C2
G.lucidum	MOL011287	lucidone A	NR3C2

G.lucidum	MOL011287	lucidone A	NCOA1
G.lucidum	MOL011309	methyl (4R)-4-[(5R,7S,10S,13R,14R,15S,17R)-7,15-dihydroxy-4,4,10,13,14-pentamethyl-3,11-dioxo-2,5,6,7,12,15,16,17-octahydro-1H-cyclopenta[a]phenanthren-17-yl]pentanoate	NR3C2
G.lucidum	MOL000279	Cerevisterol	NR3C2
G.lucidum	MOL000282	ergosta-7,22E-dien-3beta-ol	PGR
G.lucidum	MOL000358	beta-sitosterol	PGR
G.lucidum	MOL000358	beta-sitosterol	NCOA2
G.lucidum	MOL000358	beta-sitosterol	PTGS1
G.lucidum	MOL000358	beta-sitosterol	PTGS2
G.lucidum	MOL000358	beta-sitosterol	HSP90AA1
G.lucidum	MOL000358	beta-sitosterol	KCNH2
G.lucidum	MOL000358	beta-sitosterol	DRD1
G.lucidum	MOL000358	beta-sitosterol	CHRM3
G.lucidum	MOL000358	beta-sitosterol	CHRM1
G.lucidum	MOL000358	beta-sitosterol	SCN5A
G.lucidum	MOL000358	beta-sitosterol	CHRM4
G.lucidum	MOL000358	beta-sitosterol	ADRA1A
G.lucidum	MOL000358	beta-sitosterol	CHRM2
G.lucidum	MOL000358	beta-sitosterol	ADRA1B
G.lucidum	MOL000358	beta-sitosterol	ADRB2
G.lucidum	MOL000358	beta-sitosterol	CHRNA2
G.lucidum	MOL000358	beta-sitosterol	SLC6A4
G.lucidum	MOL000358	beta-sitosterol	OPRM1
G.lucidum	MOL000358	beta-sitosterol	GABRA1
G.lucidum	MOL000358	beta-sitosterol	BCL2
G.lucidum	MOL000358	beta-sitosterol	BAX
G.lucidum	MOL000358	beta-sitosterol	CASP9

G.lucidum	MOL000358	beta-sitosterol	JUN
G.lucidum	MOL000358	beta-sitosterol	CASP3
G.lucidum	MOL000358	beta-sitosterol	CASP8
G.lucidum	MOL000358	beta-sitosterol	PRKCA
G.lucidum	MOL000358	beta-sitosterol	PON1
G.lucidum	MOL000358	beta-sitosterol	MAP2
P.sinensis	MOL000211	Mairin	PGR
P.sinensis	MOL002883	Ethyl oleate (NF)	NCOA2
P.sinensis	MOL000073	ent-Epicatechin	PTGS1
P.sinensis	MOL000073	ent-Epicatechin	ESR1
P.sinensis	MOL000073	ent-Epicatechin	PTGS2
P.sinensis	MOL000073	ent-Epicatechin	HSP90AA1
P.sinensis	MOL000098	quercetin	PTGS1
P.sinensis	MOL000098	quercetin	AR
P.sinensis	MOL000098	quercetin	PPARG
P.sinensis	MOL000098	quercetin	PTGS2
P.sinensis	MOL000098	quercetin	HSP90AA1
P.sinensis	MOL000098	quercetin	NCOA2
P.sinensis	MOL000098	quercetin	DPP4
P.sinensis	MOL000098	quercetin	AKR1B1
P.sinensis	MOL000098	quercetin	PRSS1
P.sinensis	MOL000098	quercetin	HTR
P.sinensis	MOL000098	quercetin	KCNH2
P.sinensis	MOL000098	quercetin	SCN5A
P.sinensis	MOL000098	quercetin	ADRB2
P.sinensis	MOL000098	quercetin	MMP3
P.sinensis	MOL000098	quercetin	F7
P.sinensis	MOL000098	quercetin	RXRA

P.sinensis	MOL000098	quercetin	ACHE
P.sinensis	MOL000098	quercetin	GABRA1
P.sinensis	MOL000098	quercetin	MAOB
P.sinensis	MOL000098	quercetin	RELA
P.sinensis	MOL000098	quercetin	EGFR
P.sinensis	MOL000098	quercetin	AKT1
P.sinensis	MOL000098	quercetin	VEGFA
P.sinensis	MOL000098	quercetin	CCND1
P.sinensis	MOL000098	quercetin	BCL2
P.sinensis	MOL000098	quercetin	BCL2L1
P.sinensis	MOL000098	quercetin	FOS
P.sinensis	MOL000098	quercetin	CDKN1A
P.sinensis	MOL000098	quercetin	EIF6
P.sinensis	MOL000098	quercetin	BAX
P.sinensis	MOL000098	quercetin	CASP9
P.sinensis	MOL000098	quercetin	PLAU
P.sinensis	MOL000098	quercetin	MMP2
P.sinensis	MOL000098	quercetin	MMP9
P.sinensis	MOL000098	quercetin	MAPK1
P.sinensis	MOL000098	quercetin	IL10RB
P.sinensis	MOL000098	quercetin	EGF
P.sinensis	MOL000098	quercetin	RB1
P.sinensis	MOL000098	quercetin	TNFSF15
P.sinensis	MOL000098	quercetin	JUN
P.sinensis	MOL000098	quercetin	IL6
P.sinensis	MOL000098	quercetin	AHSA1
P.sinensis	MOL000098	quercetin	CASP3
P.sinensis	MOL000098	quercetin	TP63

P.sinensis	MOL000098	quercetin	ELK1
P.sinensis	MOL000098	quercetin	NFKBIA
P.sinensis	MOL000098	quercetin	POR
P.sinensis	MOL000098	quercetin	ODC1
P.sinensis	MOL000098	quercetin	CASP8
P.sinensis	MOL000098	quercetin	TOP1
P.sinensis	MOL000098	quercetin	RAF1
P.sinensis	MOL000098	quercetin	SOD1
P.sinensis	MOL000098	quercetin	PRKCA
P.sinensis	MOL000098	quercetin	MMP1
P.sinensis	MOL000098	quercetin	HIF1A
P.sinensis	MOL000098	quercetin	STAT1
P.sinensis	MOL000098	quercetin	RUNX1T1
P.sinensis	MOL000098	quercetin	CDK1
P.sinensis	MOL000098	quercetin	HSPA5
P.sinensis	MOL000098	quercetin	ERBB2
P.sinensis	MOL000098	quercetin	PPARG
P.sinensis	MOL000098	quercetin	ACACA
P.sinensis	MOL000098	quercetin	HMOX1
P.sinensis	MOL000098	quercetin	CYP3A4
P.sinensis	MOL000098	quercetin	CYP1A2
P.sinensis	MOL000098	quercetin	CAV1
P.sinensis	MOL000098	quercetin	MYC
P.sinensis	MOL000098	quercetin	F3
P.sinensis	MOL000098	quercetin	GJA1
P.sinensis	MOL000098	quercetin	CYP1A1
P.sinensis	MOL000098	quercetin	ICAM1
P.sinensis	MOL000098	quercetin	IL1B

P.sinensis	MOL000098	quercetin	CCL2
P.sinensis	MOL000098	quercetin	SELE
P.sinensis	MOL000098	quercetin	VCAM1
P.sinensis	MOL000098	quercetin	PTGER3
P.sinensis	MOL000098	quercetin	IL8RA
P.sinensis	MOL000098	quercetin	PRKCB
P.sinensis	MOL000098	quercetin	BIRC5
P.sinensis	MOL000098	quercetin	DUOX2
P.sinensis	MOL000098	quercetin	HSPB1
P.sinensis	MOL000098	quercetin	SULT1E1
P.sinensis	MOL000098	quercetin	IL2
P.sinensis	MOL000098	quercetin	NR1I2
P.sinensis	MOL000098	quercetin	CYP1B1
P.sinensis	MOL000098	quercetin	CCNB1
P.sinensis	MOL000098	quercetin	PLAT
P.sinensis	MOL000098	quercetin	THBD
P.sinensis	MOL000098	quercetin	SERPINE1
P.sinensis	MOL000098	quercetin	COL1A1
P.sinensis	MOL000098	quercetin	IFNGR1
P.sinensis	MOL000098	quercetin	ALOX5
P.sinensis	MOL000098	quercetin	IL1A
P.sinensis	MOL000098	quercetin	MPO
P.sinensis	MOL000098	quercetin	TOP2A
P.sinensis	MOL000098	quercetin	NCF1
P.sinensis	MOL000098	quercetin	ABCG2
P.sinensis	MOL000098	quercetin	HAS2
P.sinensis	MOL000098	quercetin	GSTP1
P.sinensis	MOL000098	quercetin	NFE2L2

P.sinensis	MOL000098	quercetin	NQO1
P.sinensis	MOL000098	quercetin	PARP1
P.sinensis	MOL000098	quercetin	AHR
P.sinensis	MOL000098	quercetin	PSMD3
P.sinensis	MOL000098	quercetin	COL3A1
P.sinensis	MOL000098	quercetin	CXCL11
P.sinensis	MOL000098	quercetin	CXCL2
P.sinensis	MOL000098	quercetin	DCAF5
P.sinensis	MOL000098	quercetin	NR1I3
P.sinensis	MOL000098	quercetin	CHEK2
P.sinensis	MOL000098	quercetin	INSRR
P.sinensis	MOL000098	quercetin	CLDN4
P.sinensis	MOL000098	quercetin	PPARA
P.sinensis	MOL000098	quercetin	PPARD
P.sinensis	MOL000098	quercetin	HSF1
P.sinensis	MOL000098	quercetin	CRP
P.sinensis	MOL000098	quercetin	CXCL10
P.sinensis	MOL000098	quercetin	CHUK
P.sinensis	MOL000098	quercetin	SPP1
P.sinensis	MOL000098	quercetin	RUNX2
P.sinensis	MOL000098	quercetin	RASSF1
P.sinensis	MOL000098	quercetin	E2F1
P.sinensis	MOL000098	quercetin	E2F2
P.sinensis	MOL000098	quercetin	ACPP
P.sinensis	MOL000098	quercetin	CTSD
P.sinensis	MOL000098	quercetin	IGFBP3
P.sinensis	MOL000098	quercetin	IGF2
P.sinensis	MOL000098	quercetin	CD40LG

P.sinensis	MOL000098	quercetin	IRF1
P.sinensis	MOL000098	quercetin	ERBB3
P.sinensis	MOL000098	quercetin	PON1
P.sinensis	MOL000098	quercetin	DIO1
P.sinensis	MOL000098	quercetin	PCOLCE
P.sinensis	MOL000098	quercetin	NPEPPS
P.sinensis	MOL000098	quercetin	HK2
P.sinensis	MOL000098	quercetin	RASA1
P.sinensis	MOL000098	quercetin	GSTM1
P.sinensis	MOL000098	quercetin	GSTM2
C.pinnatifida	MOL000354	isorhamnetin	PTGS1
C.pinnatifida	MOL000354	isorhamnetin	ESR1
C.pinnatifida	MOL000354	isorhamnetin	AR
C.pinnatifida	MOL000354	isorhamnetin	PPARG
C.pinnatifida	MOL000354	isorhamnetin	PTGS2
C.pinnatifida	MOL000354	isorhamnetin	ESR2
C.pinnatifida	MOL000354	isorhamnetin	DPP4
C.pinnatifida	MOL000354	isorhamnetin	MAPK14
C.pinnatifida	MOL000354	isorhamnetin	GSK3B
C.pinnatifida	MOL000354	isorhamnetin	HSP90AA1
C.pinnatifida	MOL000354	isorhamnetin	CDK2
C.pinnatifida	MOL000354	isorhamnetin	PRSS1
C.pinnatifida	MOL000354	isorhamnetin	CCNA2
C.pinnatifida	MOL000354	isorhamnetin	NCOA2
C.pinnatifida	MOL000354	isorhamnetin	CALM1
C.pinnatifida	MOL000354	isorhamnetin	PPARD
C.pinnatifida	MOL000354	isorhamnetin	CHEK1
C.pinnatifida	MOL000354	isorhamnetin	AKR1B1

C.pinnatifida	MOL000354	isorhamnetin	NCOA1
C.pinnatifida	MOL000354	isorhamnetin	F7
C.pinnatifida	MOL000354	isorhamnetin	HTR
C.pinnatifida	MOL000354	isorhamnetin	ACHE
C.pinnatifida	MOL000354	isorhamnetin	GABRA1
C.pinnatifida	MOL000354	isorhamnetin	MAOB
C.pinnatifida	MOL000354	isorhamnetin	GRIA2
C.pinnatifida	MOL000354	isorhamnetin	RELA
C.pinnatifida	MOL000354	isorhamnetin	NCF1
C.pinnatifida	MOL000354	isorhamnetin	OLR1
C.pinnatifida	MOL000359	sitosterol	PGR
C.pinnatifida	MOL000359	sitosterol	NCOA2
C.pinnatifida	MOL000359	sitosterol	NR3C2
C.pinnatifida	MOL000422	kaempferol	PTGS1
C.pinnatifida	MOL000422	kaempferol	AR
C.pinnatifida	MOL000422	kaempferol	PPARG
C.pinnatifida	MOL000422	kaempferol	PTGS2
C.pinnatifida	MOL000422	kaempferol	HSP90AA1
C.pinnatifida	MOL000422	kaempferol	NCOA2
C.pinnatifida	MOL000422	kaempferol	DPP4
C.pinnatifida	MOL000422	kaempferol	PRSS1
C.pinnatifida	MOL000422	kaempferol	PGR
C.pinnatifida	MOL000422	kaempferol	HTR
C.pinnatifida	MOL000422	kaempferol	CHRM1
C.pinnatifida	MOL000422	kaempferol	ACHE
C.pinnatifida	MOL000422	kaempferol	SLC6A2
C.pinnatifida	MOL000422	kaempferol	CHRM2
C.pinnatifida	MOL000422	kaempferol	ADRA1B

C.pinnatifida	MOL000422	kaempferol	GABRA1
C.pinnatifida	MOL000422	kaempferol	F7
C.pinnatifida	MOL000422	kaempferol	CALM1
C.pinnatifida	MOL000422	kaempferol	RELA
C.pinnatifida	MOL000422	kaempferol	IKBKB
C.pinnatifida	MOL000422	kaempferol	AKT1
C.pinnatifida	MOL000422	kaempferol	BCL2
C.pinnatifida	MOL000422	kaempferol	BAX
C.pinnatifida	MOL000422	kaempferol	TNFSF15
C.pinnatifida	MOL000422	kaempferol	JUN
C.pinnatifida	MOL000422	kaempferol	AHSA1
C.pinnatifida	MOL000422	kaempferol	CASP3
C.pinnatifida	MOL000422	kaempferol	MAPK8
C.pinnatifida	MOL000422	kaempferol	MMP1
C.pinnatifida	MOL000422	kaempferol	STAT1
C.pinnatifida	MOL000422	kaempferol	CDK1
C.pinnatifida	MOL000422	kaempferol	PPARG
C.pinnatifida	MOL000422	kaempferol	HMOX1
C.pinnatifida	MOL000422	kaempferol	CYP3A4
C.pinnatifida	MOL000422	kaempferol	CYP1A2
C.pinnatifida	MOL000422	kaempferol	CYP1A1
C.pinnatifida	MOL000422	kaempferol	ICAM1
C.pinnatifida	MOL000422	kaempferol	SELE
C.pinnatifida	MOL000422	kaempferol	VCAM1
C.pinnatifida	MOL000422	kaempferol	NR1I2
C.pinnatifida	MOL000422	kaempferol	CYP1B1
C.pinnatifida	MOL000422	kaempferol	ALOX5
C.pinnatifida	MOL000422	kaempferol	HAS2

C.pinnatifida	MOL000422	kaempferol	GSTP1
C.pinnatifida	MOL000422	kaempferol	AHR
C.pinnatifida	MOL000422	kaempferol	PSMD3
C.pinnatifida	MOL000422	kaempferol	NR1I3
C.pinnatifida	MOL000422	kaempferol	INSRR
C.pinnatifida	MOL000422	kaempferol	DIO1
C.pinnatifida	MOL000422	kaempferol	PPP3CA
C.pinnatifida	MOL000422	kaempferol	GSTM1
C.pinnatifida	MOL000422	kaempferol	GSTM2
C.pinnatifida	MOL000422	kaempferol	AKR1C3
C.pinnatifida	MOL000422	kaempferol	SLPI
C.pinnatifida	MOL000449	Stigmasterol	PGR
C.pinnatifida	MOL000449	Stigmasterol	NR3C2
C.pinnatifida	MOL000449	Stigmasterol	NCOA2
C.pinnatifida	MOL000449	Stigmasterol	ADH1C
C.pinnatifida	MOL000449	Stigmasterol	IGHG1
C.pinnatifida	MOL000449	Stigmasterol	RXRA
C.pinnatifida	MOL000449	Stigmasterol	NCOA1
C.pinnatifida	MOL000449	Stigmasterol	PTGS1
C.pinnatifida	MOL000449	Stigmasterol	PTGS2
C.pinnatifida	MOL000449	Stigmasterol	ADRA2A
C.pinnatifida	MOL000449	Stigmasterol	SLC6A2
C.pinnatifida	MOL000449	Stigmasterol	SLC6A3
C.pinnatifida	MOL000449	Stigmasterol	ADRB2
C.pinnatifida	MOL000449	Stigmasterol	AKR1B1
C.pinnatifida	MOL000449	Stigmasterol	PLAU
C.pinnatifida	MOL000449	Stigmasterol	LTA4H
C.pinnatifida	MOL000449	Stigmasterol	MAOB

C.pinnatifida	MOL000449	Stigmasterol	MAOA
C.pinnatifida	MOL000449	Stigmasterol	CTRB1
C.pinnatifida	MOL000449	Stigmasterol	CHRM3
C.pinnatifida	MOL000449	Stigmasterol	CHRM1
C.pinnatifida	MOL000449	Stigmasterol	ADRB1
C.pinnatifida	MOL000449	Stigmasterol	SCN5A
C.pinnatifida	MOL000449	Stigmasterol	ADRA1A
C.pinnatifida	MOL000449	Stigmasterol	CHRM2
C.pinnatifida	MOL000449	Stigmasterol	ADRA1B
C.pinnatifida	MOL000449	Stigmasterol	GABRA1
C.pinnatifida	MOL000073	ent-Epicatechin	PTGS1
C.pinnatifida	MOL000073	ent-Epicatechin	ESR1
C.pinnatifida	MOL000073	ent-Epicatechin	PTGS2
C.pinnatifida	MOL000073	ent-Epicatechin	HSP90AA1
C.pinnatifida	MOL000098	quercetin	PTGS1
C.pinnatifida	MOL000098	quercetin	AR
C.pinnatifida	MOL000098	quercetin	PPARG
C.pinnatifida	MOL000098	quercetin	PTGS2
C.pinnatifida	MOL000098	quercetin	HSP90AA1
C.pinnatifida	MOL000098	quercetin	NCOA2
C.pinnatifida	MOL000098	quercetin	DPP4
C.pinnatifida	MOL000098	quercetin	AKR1B1
C.pinnatifida	MOL000098	quercetin	PRSS1
C.pinnatifida	MOL000098	quercetin	HTR
C.pinnatifida	MOL000098	quercetin	KCNH2
C.pinnatifida	MOL000098	quercetin	SCN5A
C.pinnatifida	MOL000098	quercetin	ADRB2
C.pinnatifida	MOL000098	quercetin	MMP3

C.pinnatifida	MOL000098	quercetin	F7
C.pinnatifida	MOL000098	quercetin	RXRA
C.pinnatifida	MOL000098	quercetin	ACHE
C.pinnatifida	MOL000098	quercetin	GABRA1
C.pinnatifida	MOL000098	quercetin	MAOB
C.pinnatifida	MOL000098	quercetin	RELA
C.pinnatifida	MOL000098	quercetin	EGFR
C.pinnatifida	MOL000098	quercetin	AKT1
C.pinnatifida	MOL000098	quercetin	VEGFA
C.pinnatifida	MOL000098	quercetin	CCND1
C.pinnatifida	MOL000098	quercetin	BCL2
C.pinnatifida	MOL000098	quercetin	BCL2L1
C.pinnatifida	MOL000098	quercetin	FOS
C.pinnatifida	MOL000098	quercetin	CDKN1A
C.pinnatifida	MOL000098	quercetin	EIF6
C.pinnatifida	MOL000098	quercetin	BAX
C.pinnatifida	MOL000098	quercetin	CASP9
C.pinnatifida	MOL000098	quercetin	PLAU
C.pinnatifida	MOL000098	quercetin	MMP2
C.pinnatifida	MOL000098	quercetin	MMP9
C.pinnatifida	MOL000098	quercetin	MAPK1
C.pinnatifida	MOL000098	quercetin	IL10RB
C.pinnatifida	MOL000098	quercetin	EGF
C.pinnatifida	MOL000098	quercetin	RB1
C.pinnatifida	MOL000098	quercetin	TNFSF15
C.pinnatifida	MOL000098	quercetin	JUN
C.pinnatifida	MOL000098	quercetin	IL6
C.pinnatifida	MOL000098	quercetin	AHSA1

C.pinnatifida	MOL000098	quercetin	CASP3
C.pinnatifida	MOL000098	quercetin	TP63
C.pinnatifida	MOL000098	quercetin	ELK1
C.pinnatifida	MOL000098	quercetin	NFKBIA
C.pinnatifida	MOL000098	quercetin	POR
C.pinnatifida	MOL000098	quercetin	ODC1
C.pinnatifida	MOL000098	quercetin	CASP8
C.pinnatifida	MOL000098	quercetin	TOP1
C.pinnatifida	MOL000098	quercetin	RAF1
C.pinnatifida	MOL000098	quercetin	SOD1
C.pinnatifida	MOL000098	quercetin	PRKCA
C.pinnatifida	MOL000098	quercetin	MMP1
C.pinnatifida	MOL000098	quercetin	HIF1A
C.pinnatifida	MOL000098	quercetin	STAT1
C.pinnatifida	MOL000098	quercetin	RUNX1T1
C.pinnatifida	MOL000098	quercetin	CDK1
C.pinnatifida	MOL000098	quercetin	HSPA5
C.pinnatifida	MOL000098	quercetin	ERBB2
C.pinnatifida	MOL000098	quercetin	PPARG
C.pinnatifida	MOL000098	quercetin	ACACA
C.pinnatifida	MOL000098	quercetin	HMOX1
C.pinnatifida	MOL000098	quercetin	CYP3A4
C.pinnatifida	MOL000098	quercetin	CYP1A2
C.pinnatifida	MOL000098	quercetin	CAV1
C.pinnatifida	MOL000098	quercetin	MYC
C.pinnatifida	MOL000098	quercetin	F3
C.pinnatifida	MOL000098	quercetin	GJA1
C.pinnatifida	MOL000098	quercetin	CYP1A1

C.pinnatifida	MOL000098	quercetin	ICAM1
C.pinnatifida	MOL000098	quercetin	IL1B
C.pinnatifida	MOL000098	quercetin	CCL2
C.pinnatifida	MOL000098	quercetin	SELE
C.pinnatifida	MOL000098	quercetin	VCAM1
C.pinnatifida	MOL000098	quercetin	PTGER3
C.pinnatifida	MOL000098	quercetin	IL8RA
C.pinnatifida	MOL000098	quercetin	PRKCB
C.pinnatifida	MOL000098	quercetin	BIRC5
C.pinnatifida	MOL000098	quercetin	DUOX2
C.pinnatifida	MOL000098	quercetin	HSPB1
C.pinnatifida	MOL000098	quercetin	SULT1E1
C.pinnatifida	MOL000098	quercetin	IL2
C.pinnatifida	MOL000098	quercetin	NR1I2
C.pinnatifida	MOL000098	quercetin	CYP1B1
C.pinnatifida	MOL000098	quercetin	CCNB1
C.pinnatifida	MOL000098	quercetin	PLAT
C.pinnatifida	MOL000098	quercetin	THBD
C.pinnatifida	MOL000098	quercetin	SERPINE1
C.pinnatifida	MOL000098	quercetin	COL1A1
C.pinnatifida	MOL000098	quercetin	IFNGR1
C.pinnatifida	MOL000098	quercetin	ALOX5
C.pinnatifida	MOL000098	quercetin	IL1A
C.pinnatifida	MOL000098	quercetin	MPO
C.pinnatifida	MOL000098	quercetin	TOP2A
C.pinnatifida	MOL000098	quercetin	NCF1
C.pinnatifida	MOL000098	quercetin	ABCG2
C.pinnatifida	MOL000098	quercetin	HAS2

C.pinnatifida	MOL000098	quercetin	GSTP1
C.pinnatifida	MOL000098	quercetin	NFE2L2
C.pinnatifida	MOL000098	quercetin	NQO1
C.pinnatifida	MOL000098	quercetin	PARP1
C.pinnatifida	MOL000098	quercetin	AHR
C.pinnatifida	MOL000098	quercetin	PSMD3
C.pinnatifida	MOL000098	quercetin	COL3A1
C.pinnatifida	MOL000098	quercetin	CXCL11
C.pinnatifida	MOL000098	quercetin	CXCL2
C.pinnatifida	MOL000098	quercetin	DCAF5
C.pinnatifida	MOL000098	quercetin	NR1I3
C.pinnatifida	MOL000098	quercetin	CHEK2
C.pinnatifida	MOL000098	quercetin	INSRR
C.pinnatifida	MOL000098	quercetin	CLDN4
C.pinnatifida	MOL000098	quercetin	PPARA
C.pinnatifida	MOL000098	quercetin	PPARD
C.pinnatifida	MOL000098	quercetin	HSF1
C.pinnatifida	MOL000098	quercetin	CRP
C.pinnatifida	MOL000098	quercetin	CXCL10
C.pinnatifida	MOL000098	quercetin	CHUK
C.pinnatifida	MOL000098	quercetin	SPP1
C.pinnatifida	MOL000098	quercetin	RUNX2
C.pinnatifida	MOL000098	quercetin	RASSF1
C.pinnatifida	MOL000098	quercetin	E2F1
C.pinnatifida	MOL000098	quercetin	E2F2
C.pinnatifida	MOL000098	quercetin	ACPP
C.pinnatifida	MOL000098	quercetin	CTSD
C.pinnatifida	MOL000098	quercetin	IGFBP3

C.pinnatifida	MOL000098	quercetin	IGF2
C.pinnatifida	MOL000098	quercetin	CD40LG
C.pinnatifida	MOL000098	quercetin	IRF1
C.pinnatifida	MOL000098	quercetin	ERBB3
C.pinnatifida	MOL000098	quercetin	PON1
C.pinnatifida	MOL000098	quercetin	DIO1
C.pinnatifida	MOL000098	quercetin	PCOLCE
C.pinnatifida	MOL000098	quercetin	NPEPPS
C.pinnatifida	MOL000098	quercetin	HK2
C.pinnatifida	MOL000098	quercetin	RASA1
C.pinnatifida	MOL000098	quercetin	GSTM1
C.pinnatifida	MOL000098	quercetin	GSTM2
H.dulcis	MOL002140	Perlolyrine	HTR
H.dulcis	MOL002140	Perlolyrine	PTGS2
H.dulcis	MOL002140	Perlolyrine	RXRA
H.dulcis	MOL000358	beta-sitosterol	PGR
H.dulcis	MOL000358	beta-sitosterol	NCOA2
H.dulcis	MOL000358	beta-sitosterol	PTGS1
H.dulcis	MOL000358	beta-sitosterol	PTGS2
H.dulcis	MOL000358	beta-sitosterol	HSP90AA1
H.dulcis	MOL000358	beta-sitosterol	KCNH2
H.dulcis	MOL000358	beta-sitosterol	DRD1
H.dulcis	MOL000358	beta-sitosterol	CHRM3
H.dulcis	MOL000358	beta-sitosterol	CHRM1
H.dulcis	MOL000358	beta-sitosterol	SCN5A
H.dulcis	MOL000358	beta-sitosterol	CHRM4
H.dulcis	MOL000358	beta-sitosterol	ADRA1A
H.dulcis	MOL000358	beta-sitosterol	CHRM2

H.dulcis	MOL000358	beta-sitosterol	ADRA1B
H.dulcis	MOL000358	beta-sitosterol	ADRB2
H.dulcis	MOL000358	beta-sitosterol	CHRNA2
H.dulcis	MOL000358	beta-sitosterol	SLC6A4
H.dulcis	MOL000358	beta-sitosterol	OPRM1
H.dulcis	MOL000358	beta-sitosterol	GABRA1
H.dulcis	MOL000358	beta-sitosterol	BCL2
H.dulcis	MOL000358	beta-sitosterol	BAX
H.dulcis	MOL000358	beta-sitosterol	CASP9
H.dulcis	MOL000358	beta-sitosterol	JUN
H.dulcis	MOL000358	beta-sitosterol	CASP3
H.dulcis	MOL000358	beta-sitosterol	CASP8
H.dulcis	MOL000358	beta-sitosterol	PRKCA
H.dulcis	MOL000358	beta-sitosterol	PON1
H.dulcis	MOL000358	beta-sitosterol	MAP2
H.dulcis	MOL000422	kaempferol	PTGS1
H.dulcis	MOL000422	kaempferol	AR
H.dulcis	MOL000422	kaempferol	PPARG
H.dulcis	MOL000422	kaempferol	PTGS2
H.dulcis	MOL000422	kaempferol	HSP90AA1
H.dulcis	MOL000422	kaempferol	NCOA2
H.dulcis	MOL000422	kaempferol	DPP4
H.dulcis	MOL000422	kaempferol	PRSS1
H.dulcis	MOL000422	kaempferol	PGR
H.dulcis	MOL000422	kaempferol	HTR
H.dulcis	MOL000422	kaempferol	CHRM1
H.dulcis	MOL000422	kaempferol	ACHE
H.dulcis	MOL000422	kaempferol	SLC6A2

H.dulcis	MOL000422	kaempferol	CHRM2
H.dulcis	MOL000422	kaempferol	ADRA1B
H.dulcis	MOL000422	kaempferol	GABRA1
H.dulcis	MOL000422	kaempferol	F7
H.dulcis	MOL000422	kaempferol	CALM1
H.dulcis	MOL000422	kaempferol	RELA
H.dulcis	MOL000422	kaempferol	IKBKB
H.dulcis	MOL000422	kaempferol	AKT1
H.dulcis	MOL000422	kaempferol	BCL2
H.dulcis	MOL000422	kaempferol	BAX
H.dulcis	MOL000422	kaempferol	TNFSF15
H.dulcis	MOL000422	kaempferol	JUN
H.dulcis	MOL000422	kaempferol	AHSA1
H.dulcis	MOL000422	kaempferol	CASP3
H.dulcis	MOL000422	kaempferol	MAPK8
H.dulcis	MOL000422	kaempferol	MMP1
H.dulcis	MOL000422	kaempferol	STAT1
H.dulcis	MOL000422	kaempferol	CDK1
H.dulcis	MOL000422	kaempferol	PPARG
H.dulcis	MOL000422	kaempferol	HMOX1
H.dulcis	MOL000422	kaempferol	CYP3A4
H.dulcis	MOL000422	kaempferol	CYP1A2
H.dulcis	MOL000422	kaempferol	CYP1A1
H.dulcis	MOL000422	kaempferol	ICAM1
H.dulcis	MOL000422	kaempferol	SELE
H.dulcis	MOL000422	kaempferol	VCAM1
H.dulcis	MOL000422	kaempferol	NR1I2
H.dulcis	MOL000422	kaempferol	CYP1B1

H.dulcis	MOL000422	kaempferol	ALOX5
H.dulcis	MOL000422	kaempferol	HAS2
H.dulcis	MOL000422	kaempferol	GSTP1
H.dulcis	MOL000422	kaempferol	AHR
H.dulcis	MOL000422	kaempferol	PSMD3
H.dulcis	MOL000422	kaempferol	NR1I3
H.dulcis	MOL000422	kaempferol	INSRR
H.dulcis	MOL000422	kaempferol	DIO1
H.dulcis	MOL000422	kaempferol	PPP3CA
H.dulcis	MOL000422	kaempferol	GSTM1
H.dulcis	MOL000422	kaempferol	GSTM2
H.dulcis	MOL000422	kaempferol	AKR1C3
H.dulcis	MOL000422	kaempferol	SLPI
H.dulcis	MOL004328	naringenin	PTGS1
H.dulcis	MOL004328	naringenin	ESR1
H.dulcis	MOL004328	naringenin	PTGS2
H.dulcis	MOL004328	naringenin	HSP90AA1
H.dulcis	MOL004328	naringenin	RELA
H.dulcis	MOL004328	naringenin	AKT1
H.dulcis	MOL004328	naringenin	BCL2
H.dulcis	MOL004328	naringenin	MAPK3
H.dulcis	MOL004328	naringenin	MAPK1
H.dulcis	MOL004328	naringenin	CASP3
H.dulcis	MOL004328	naringenin	FASN
H.dulcis	MOL004328	naringenin	LDLR
H.dulcis	MOL004328	naringenin	BAD
H.dulcis	MOL004328	naringenin	SOD1
H.dulcis	MOL004328	naringenin	CAT

H.dulcis	MOL004328	naringenin	PPARG
H.dulcis	MOL004328	naringenin	MTTP
H.dulcis	MOL004328	naringenin	APOB
H.dulcis	MOL004328	naringenin	HMGCR
H.dulcis	MOL004328	naringenin	CYP19A1
H.dulcis	MOL004328	naringenin	GSTP1
H.dulcis	MOL004328	naringenin	UGT1A1
H.dulcis	MOL004328	naringenin	PPARA
H.dulcis	MOL004328	naringenin	SREBF1
H.dulcis	MOL004328	naringenin	ABCC1
H.dulcis	MOL004328	naringenin	C1QB
H.dulcis	MOL004328	naringenin	SOAT2
H.dulcis	MOL004328	naringenin	AKR1C1
H.dulcis	MOL004328	naringenin	CES1
H.dulcis	MOL004328	naringenin	SOAT1
H.dulcis	MOL000449	Stigmasterol	PGR
H.dulcis	MOL000449	Stigmasterol	NR3C2
H.dulcis	MOL000449	Stigmasterol	NCOA2
H.dulcis	MOL000449	Stigmasterol	ADH1C
H.dulcis	MOL000449	Stigmasterol	IGHG1
H.dulcis	MOL000449	Stigmasterol	RXRA
H.dulcis	MOL000449	Stigmasterol	NCOA1
H.dulcis	MOL000449	Stigmasterol	PTGS1
H.dulcis	MOL000449	Stigmasterol	PTGS2
H.dulcis	MOL000449	Stigmasterol	ADRA2A
H.dulcis	MOL000449	Stigmasterol	SLC6A2
H.dulcis	MOL000449	Stigmasterol	SLC6A3
H.dulcis	MOL000449	Stigmasterol	ADRB2

H.dulcis	MOL000449	Stigmasterol	AKR1B1
H.dulcis	MOL000449	Stigmasterol	PLAU
H.dulcis	MOL000449	Stigmasterol	LTA4H
H.dulcis	MOL000449	Stigmasterol	MAOB
H.dulcis	MOL000449	Stigmasterol	MAOA
H.dulcis	MOL000449	Stigmasterol	CTRB1
H.dulcis	MOL000449	Stigmasterol	CHRM3
H.dulcis	MOL000449	Stigmasterol	CHRM1
H.dulcis	MOL000449	Stigmasterol	ADRB1
H.dulcis	MOL000449	Stigmasterol	SCN5A
H.dulcis	MOL000449	Stigmasterol	ADRA1A
H.dulcis	MOL000449	Stigmasterol	CHRM2
H.dulcis	MOL000449	Stigmasterol	ADRA1B
H.dulcis	MOL000449	Stigmasterol	GABRA1
H.dulcis	MOL000098	quercetin	PTGS1
H.dulcis	MOL000098	quercetin	AR
H.dulcis	MOL000098	quercetin	PPARG
H.dulcis	MOL000098	quercetin	PTGS2
H.dulcis	MOL000098	quercetin	HSP90AA1
H.dulcis	MOL000098	quercetin	NCOA2
H.dulcis	MOL000098	quercetin	DPP4
H.dulcis	MOL000098	quercetin	AKR1B1
H.dulcis	MOL000098	quercetin	PRSS1
H.dulcis	MOL000098	quercetin	HTR
H.dulcis	MOL000098	quercetin	KCNH2
H.dulcis	MOL000098	quercetin	SCN5A
H.dulcis	MOL000098	quercetin	ADRB2
H.dulcis	MOL000098	quercetin	MMP3

H.dulcis	MOL000098	quercetin	F7
H.dulcis	MOL000098	quercetin	RXRA
H.dulcis	MOL000098	quercetin	ACHE
H.dulcis	MOL000098	quercetin	GABRA1
H.dulcis	MOL000098	quercetin	MAOB
H.dulcis	MOL000098	quercetin	RELA
H.dulcis	MOL000098	quercetin	EGFR
H.dulcis	MOL000098	quercetin	AKT1
H.dulcis	MOL000098	quercetin	VEGFA
H.dulcis	MOL000098	quercetin	CCND1
H.dulcis	MOL000098	quercetin	BCL2
H.dulcis	MOL000098	quercetin	BCL2L1
H.dulcis	MOL000098	quercetin	FOS
H.dulcis	MOL000098	quercetin	CDKN1A
H.dulcis	MOL000098	quercetin	EIF6
H.dulcis	MOL000098	quercetin	BAX
H.dulcis	MOL000098	quercetin	CASP9
H.dulcis	MOL000098	quercetin	PLAU
H.dulcis	MOL000098	quercetin	MMP2
H.dulcis	MOL000098	quercetin	MMP9
H.dulcis	MOL000098	quercetin	MAPK1
H.dulcis	MOL000098	quercetin	IL10RB
H.dulcis	MOL000098	quercetin	EGF
H.dulcis	MOL000098	quercetin	RB1
H.dulcis	MOL000098	quercetin	TNFSF15
H.dulcis	MOL000098	quercetin	JUN
H.dulcis	MOL000098	quercetin	IL6
H.dulcis	MOL000098	quercetin	AHSA1

H.dulcis	MOL000098	quercetin	CASP3
H.dulcis	MOL000098	quercetin	TP63
H.dulcis	MOL000098	quercetin	ELK1
H.dulcis	MOL000098	quercetin	NFKBIA
H.dulcis	MOL000098	quercetin	POR
H.dulcis	MOL000098	quercetin	ODC1
H.dulcis	MOL000098	quercetin	CASP8
H.dulcis	MOL000098	quercetin	TOP1
H.dulcis	MOL000098	quercetin	RAF1
H.dulcis	MOL000098	quercetin	SOD1
H.dulcis	MOL000098	quercetin	PRKCA
H.dulcis	MOL000098	quercetin	MMP1
H.dulcis	MOL000098	quercetin	HIF1A
H.dulcis	MOL000098	quercetin	STAT1
H.dulcis	MOL000098	quercetin	RUNX1T1
H.dulcis	MOL000098	quercetin	CDK1
H.dulcis	MOL000098	quercetin	HSPA5
H.dulcis	MOL000098	quercetin	ERBB2
H.dulcis	MOL000098	quercetin	PPARG
H.dulcis	MOL000098	quercetin	ACACA
H.dulcis	MOL000098	quercetin	HMOX1
H.dulcis	MOL000098	quercetin	CYP3A4
H.dulcis	MOL000098	quercetin	CYP1A2
H.dulcis	MOL000098	quercetin	CAV1
H.dulcis	MOL000098	quercetin	MYC
H.dulcis	MOL000098	quercetin	F3
H.dulcis	MOL000098	quercetin	GJA1
H.dulcis	MOL000098	quercetin	CYP1A1

H.dulcis	MOL000098	quercetin	ICAM1
H.dulcis	MOL000098	quercetin	IL1B
H.dulcis	MOL000098	quercetin	CCL2
H.dulcis	MOL000098	quercetin	SELE
H.dulcis	MOL000098	quercetin	VCAM1
H.dulcis	MOL000098	quercetin	PTGER3
H.dulcis	MOL000098	quercetin	IL8RA
H.dulcis	MOL000098	quercetin	PRKCB
H.dulcis	MOL000098	quercetin	BIRC5
H.dulcis	MOL000098	quercetin	DUOX2
H.dulcis	MOL000098	quercetin	HSPB1
H.dulcis	MOL000098	quercetin	SULT1E1
H.dulcis	MOL000098	quercetin	IL2
H.dulcis	MOL000098	quercetin	NR1I2
H.dulcis	MOL000098	quercetin	CYP1B1
H.dulcis	MOL000098	quercetin	CCNB1
H.dulcis	MOL000098	quercetin	PLAT
H.dulcis	MOL000098	quercetin	THBD
H.dulcis	MOL000098	quercetin	SERPINE1
H.dulcis	MOL000098	quercetin	COL1A1
H.dulcis	MOL000098	quercetin	IFNGR1
H.dulcis	MOL000098	quercetin	ALOX5
H.dulcis	MOL000098	quercetin	IL1A
H.dulcis	MOL000098	quercetin	MPO
H.dulcis	MOL000098	quercetin	TOP2A
H.dulcis	MOL000098	quercetin	NCF1
H.dulcis	MOL000098	quercetin	ABCG2
H.dulcis	MOL000098	quercetin	HAS2

H.dulcis	MOL000098	quercetin	GSTP1
H.dulcis	MOL000098	quercetin	NFE2L2
H.dulcis	MOL000098	quercetin	NQO1
H.dulcis	MOL000098	quercetin	PARP1
H.dulcis	MOL000098	quercetin	AHR
H.dulcis	MOL000098	quercetin	PSMD3
H.dulcis	MOL000098	quercetin	COL3A1
H.dulcis	MOL000098	quercetin	CXCL11
H.dulcis	MOL000098	quercetin	CXCL2
H.dulcis	MOL000098	quercetin	DCAF5
H.dulcis	MOL000098	quercetin	NR1I3
H.dulcis	MOL000098	quercetin	CHEK2
H.dulcis	MOL000098	quercetin	INSRR
H.dulcis	MOL000098	quercetin	CLDN4
H.dulcis	MOL000098	quercetin	PPARA
H.dulcis	MOL000098	quercetin	PPARD
H.dulcis	MOL000098	quercetin	HSF1
H.dulcis	MOL000098	quercetin	CRP
H.dulcis	MOL000098	quercetin	CXCL10
H.dulcis	MOL000098	quercetin	CHUK
H.dulcis	MOL000098	quercetin	SPP1
H.dulcis	MOL000098	quercetin	RUNX2
H.dulcis	MOL000098	quercetin	RASSF1
H.dulcis	MOL000098	quercetin	E2F1
H.dulcis	MOL000098	quercetin	E2F2
H.dulcis	MOL000098	quercetin	ACPP
H.dulcis	MOL000098	quercetin	CTSD
H.dulcis	MOL000098	quercetin	IGFBP3

H.dulcis	MOL000098	quercetin	IGF2
H.dulcis	MOL000098	quercetin	CD40LG
H.dulcis	MOL000098	quercetin	IRF1
H.dulcis	MOL000098	quercetin	ERBB3
H.dulcis	MOL000098	quercetin	PON1
H.dulcis	MOL000098	quercetin	DIO1
H.dulcis	MOL000098	quercetin	PCOLCE
H.dulcis	MOL000098	quercetin	NPEPPS
H.dulcis	MOL000098	quercetin	HK2
H.dulcis	MOL000098	quercetin	RASA1
H.dulcis	MOL000098	quercetin	GSTM1
H.dulcis	MOL000098	quercetin	GSTM2
M.pupureus	MOL000449	Stigmasterol	PGR
M.pupureus	MOL000449	Stigmasterol	NR3C2
M.pupureus	MOL000449	Stigmasterol	NCOA2
M.pupureus	MOL000449	Stigmasterol	ADH1C
M.pupureus	MOL000449	Stigmasterol	IGHG1
M.pupureus	MOL000449	Stigmasterol	RXRA
M.pupureus	MOL000449	Stigmasterol	NCOA1
M.pupureus	MOL000449	Stigmasterol	PTGS1
M.pupureus	MOL000449	Stigmasterol	PTGS2
M.pupureus	MOL000449	Stigmasterol	ADRA2A
M.pupureus	MOL000449	Stigmasterol	SLC6A2
M.pupureus	MOL000449	Stigmasterol	SLC6A3
M.pupureus	MOL000449	Stigmasterol	ADRB2
M.pupureus	MOL000449	Stigmasterol	AKR1B1
M.pupureus	MOL000449	Stigmasterol	PLAU
M.pupureus	MOL000449	Stigmasterol	LTA4H

M.pupureus	MOL000449	Stigmasterol	MAOB
M.pupureus	MOL000449	Stigmasterol	MAOA
M.pupureus	MOL000449	Stigmasterol	CTRB1
M.pupureus	MOL000449	Stigmasterol	CHRM3
M.pupureus	MOL000449	Stigmasterol	CHRM1
M.pupureus	MOL000449	Stigmasterol	ADRB1
M.pupureus	MOL000449	Stigmasterol	SCN5A
M.pupureus	MOL000449	Stigmasterol	ADRA1A
M.pupureus	MOL000449	Stigmasterol	CHRM2
M.pupureus	MOL000449	Stigmasterol	ADRA1B
M.pupureus	MOL000449	Stigmasterol	GABRA1
M.pupureus	MOL000298	Ergosterol	AR
M.pupureus	MOL000298	Ergosterol	PGR
M.pupureus	MOL000298	Ergosterol	NR3C1
M.pupureus	MOL000298	Ergosterol	NCOA2
M.pupureus	MOL000298	Ergosterol	DHCR7
M.pupureus	MOL000388	gamma-aminobutyric acid	CTSD
M.pupureus	MOL000388	gamma-aminobutyric acid	BCHE
M.pupureus	MOL000388	gamma-aminobutyric acid	ADH1B
M.pupureus	MOL000388	gamma-aminobutyric acid	ADH1C
M.pupureus	MOL000388	gamma-aminobutyric acid	GOT2
M.pupureus	MOL000388	gamma-aminobutyric acid	PRSS3
M.pupureus	MOL000388	gamma-aminobutyric acid	ADH1A
M.pupureus	MOL000388	gamma-aminobutyric acid	IL6
M.pupureus	MOL000388	gamma-aminobutyric acid	VCP
M.pupureus	MOL000388	gamma-aminobutyric acid	SLC6A1
M.pupureus	MOL000481	genistein	PTGS1
M.pupureus	MOL000481	genistein	ESR1

M.pupureus	MOL000481	genistein	PPARG
M.pupureus	MOL000481	genistein	PTGS2
M.pupureus	MOL000481	genistein	MAPK14
M.pupureus	MOL000481	genistein	HSP90AA1
M.pupureus	MOL000481	genistein	CDK2
M.pupureus	MOL000481	genistein	CHEK1
M.pupureus	MOL000481	genistein	PRSS1
M.pupureus	MOL000481	genistein	RELA
M.pupureus	MOL000481	genistein	EGFR
M.pupureus	MOL000481	genistein	STAT3
M.pupureus	MOL000481	genistein	AKT1
M.pupureus	MOL000481	genistein	VEGFA
M.pupureus	MOL000481	genistein	BCL2
M.pupureus	MOL000481	genistein	FOS
M.pupureus	MOL000481	genistein	CDKN1A
M.pupureus	MOL000481	genistein	BAX
M.pupureus	MOL000481	genistein	CASP9
M.pupureus	MOL000481	genistein	MMP9
M.pupureus	MOL000481	genistein	MAPK3
M.pupureus	MOL000481	genistein	MAPK1
M.pupureus	MOL000481	genistein	TNFSF15
M.pupureus	MOL000481	genistein	JUN
M.pupureus	MOL000481	genistein	AHSA1
M.pupureus	MOL000481	genistein	CASP3
M.pupureus	MOL000481	genistein	TP63
M.pupureus	MOL000481	genistein	LDLR
M.pupureus	MOL000481	genistein	MDM2
M.pupureus	MOL000481	genistein	RASGRF2

M.pupureus	MOL000481	genistein	RAF1
M.pupureus	MOL000481	genistein	TEP1
M.pupureus	MOL000481	genistein	TIMP1
M.pupureus	MOL000481	genistein	HIF1A
M.pupureus	MOL000481	genistein	IGF1R
M.pupureus	MOL000481	genistein	STAT1
M.pupureus	MOL000481	genistein	CDK1
M.pupureus	MOL000481	genistein	ERBB2
M.pupureus	MOL000481	genistein	AR
M.pupureus	MOL000481	genistein	PPARG
M.pupureus	MOL000481	genistein	ICAM1
M.pupureus	MOL000481	genistein	IL1B
M.pupureus	MOL000481	genistein	CCL2
M.pupureus	MOL000481	genistein	SELE
M.pupureus	MOL000481	genistein	VCAM1
M.pupureus	MOL000481	genistein	FSD1
M.pupureus	MOL000481	genistein	IL8RA
M.pupureus	MOL000481	genistein	BIRC5
M.pupureus	MOL000481	genistein	SULT1E1
M.pupureus	MOL000481	genistein	CCNB1
M.pupureus	MOL000481	genistein	CDC25C
M.pupureus	MOL000481	genistein	HMGCR
M.pupureus	MOL000481	genistein	BTK
M.pupureus	MOL000481	genistein	PLK2
M.pupureus	MOL000481	genistein	GFAP
M.pupureus	MOL000481	genistein	UGT1A1
M.pupureus	MOL000481	genistein	CHEK2
M.pupureus	MOL000481	genistein	PPARA

M.pupureus	MOL000481	genistein	PCOLCE
M.pupureus	MOL000481	genistein	ATM
M.pupureus	MOL000481	genistein	INS
M.pupureus	MOL000481	genistein	PBK
M.pupureus	MOL000481	genistein	BUB1
M.pupureus	MOL000481	genistein	CDC20
M.pupureus	MOL000481	genistein	AKR1B1
M.pupureus	MOL000481	genistein	CRYAB
M.pupureus	MOL000481	genistein	KLK3
M.pupureus	MOL000481	genistein	CDC37
M.pupureus	MOL000481	genistein	HDAC6
M.pupureus	MOL000481	genistein	MAP2K5
M.pupureus	MOL000481	genistein	MAPK12
M.pupureus	MOL000481	genistein	APOA1
M.pupureus	MOL000481	genistein	PLK1
M.pupureus	MOL000481	genistein	CCNB1IP1
M.pupureus	MOL000481	genistein	MDC1
M.pupureus	MOL000481	genistein	SAFB
M.pupureus	MOL000481	genistein	CFTR
M.pupureus	MOL000481	genistein	NCOR1
M.pupureus	MOL000481	genistein	GCKR
M.pupureus	MOL000481	genistein	KCNJ11
M.pupureus	MOL000481	genistein	PTGER2
M.pupureus	MOL000481	genistein	PTGFR
M.pupureus	MOL000481	genistein	GLB1
M.pupureus	MOL000481	genistein	MSLN
M.pupureus	MOL000481	genistein	SYK
M.pupureus	MOL000390	daidzein	PTGS1

M.pupureus	MOL000390	daidzein	ESR1
M.pupureus	MOL000390	daidzein	PPARG
M.pupureus	MOL000390	daidzein	PTGS2
M.pupureus	MOL000390	daidzein	RXRA
M.pupureus	MOL000390	daidzein	ADRB2
M.pupureus	MOL000390	daidzein	MAPK14
M.pupureus	MOL000390	daidzein	HSP90AA1
M.pupureus	MOL000390	daidzein	CDK2
M.pupureus	MOL000390	daidzein	CHEK1
M.pupureus	MOL000390	daidzein	PRSS1
M.pupureus	MOL000390	daidzein	RELA
M.pupureus	MOL000390	daidzein	VEGFA
M.pupureus	MOL000390	daidzein	FOS
M.pupureus	MOL000390	daidzein	CDKN1A
M.pupureus	MOL000390	daidzein	EIF6
M.pupureus	MOL000390	daidzein	BAX
M.pupureus	MOL000390	daidzein	TNFSF15
M.pupureus	MOL000390	daidzein	JUN
M.pupureus	MOL000390	daidzein	IL6
M.pupureus	MOL000390	daidzein	CASP3
M.pupureus	MOL000390	daidzein	TP63
M.pupureus	MOL000390	daidzein	LDLR
M.pupureus	MOL000390	daidzein	CAT
M.pupureus	MOL000390	daidzein	IGF1R
M.pupureus	MOL000390	daidzein	STAT1
M.pupureus	MOL000390	daidzein	PPARG
M.pupureus	MOL000390	daidzein	CYP3A4
M.pupureus	MOL000390	daidzein	CAV1

M.pupureus	MOL000390	daidzein	ICAM1
M.pupureus	MOL000390	daidzein	MTTP
M.pupureus	MOL000390	daidzein	APOB
M.pupureus	MOL000390	daidzein	VCAM1
M.pupureus	MOL000390	daidzein	ECE1
M.pupureus	MOL000390	daidzein	GABBR1
M.pupureus	MOL000390	daidzein	IL4
M.pupureus	MOL000390	daidzein	CALM1
M.pupureus	MOL000390	daidzein	AHR
M.pupureus	MOL000390	daidzein	RHOA
M.pupureus	MOL000390	daidzein	MT2A
M.pupureus	MOL000390	daidzein	FCER2
M.pupureus	MOL000390	daidzein	MKI67
M.pupureus	MOL000390	daidzein	EP300
M.pupureus	MOL000390	daidzein	ND6
M.pupureus	MOL000390	daidzein	HSD3B2
M.pupureus	MOL000390	daidzein	HSD3B1
M.pupureus	MOL000390	daidzein	TFF1
M.pupureus	MOL000390	daidzein	TRPM2
M.pupureus	MOL000390	daidzein	GH1
M.pupureus	MOL000390	daidzein	PPARGC1B
M.pupureus	MOL000390	daidzein	GHR
M.pupureus	MOL000390	daidzein	TGFB1I1
M.pupureus	MOL000390	daidzein	CD5L
M.pupureus	MOL000390	daidzein	P4HB
M.pupureus	MOL000390	daidzein	CYP21A2
M.pupureus	MOL000390	daidzein	GADD45A
M.pupureus	MOL000390	daidzein	BARD1

M.pupureus	MOL000390	daidzein	RBM45
M.pupureus	MOL000390	daidzein	BAP1
M.pupureus	MOL000390	daidzein	RAD51

Table SII: Nonalcoholic fatty liver targets.

Nonalcoholic fatty liver targets
GSTM1
SRSF11
CP
CP
FGG
CD1D1
TRP53INP1
MFSD2A
MFSD2A
RSRP1
ERRFI1
PNISR
STRA6L
H3F3B
ASL
GCKR
PCYT2
AFM
AFM
AASS
AASS
AASS

APOE
NUDT7
SLC25A15
LACTB2
HERPUD1
HIGD1A
CFH
FDX1
FAM214A
SAT1
SAT1
SSR4
SLC19A2
FH1
FH1
VMP1
SERPINA3K
SERPINA3N
SLC25A47
SLC25A47
SLC25A47
IFRD1
IFRD1
IFRD1
IFRD1
APOB
SRSF5
TUBB2A

TUBB2A
CTSL
CTSL
CTSL
ACOT12
ITIH1
GLUD1
KRT8
KRT8
RETREG1
AOX3
FTCD
EIF4A2
EIF4A2
EIF4A2
CPS1
TCP1
TCP1
TCP1
TCP1
TCP1
DNAJB2
MYL12A
LPIN2
LPIN2
LPIN2
LPIN2
LPIN2

ZFAND5
GOT1
GOT1
SCD1
SLC22A30
GANAB
DSTN
HC
MCM10
ABCD3
DHCR24
DECR1
SDS
POR
GNB2
CROT
RPN1
CTSD
TMEM147
AMT
HMBS
APOC3
ACADVL
KIF1C
APOB
CYC1
CYP2D10
STUB1

BRD2
HSP90AB1
C3
C3
C3
C3
ACY3
SLC25A3
SYVN1
SNRNP200
IDH3B
CTSA
MUTUALLY EXCLUSIVE EXONS
GENE
H2AFY
ALTERNATIVE 5' SPLICE SITES
GENE
UBE2D3
LARP1B
AADAC
ARHGEF11
CERS2
MIER1
PAFAH2
RPL38
EIF4G3
FGGY
NUDT9

NONE
SDS
MLXIPL
5830473C10RIK
5830473C10RIK
PPARG
ST3GAL5
ST3GAL5
EVA1A
MGLL
ALDH1L1
RPS19
COX4I1
NONE
ADIPOR1
GPT2
SEC14L4
APOA5
FAM214A
IKBKG
TMEM256
PFDN2
PSMD11
TAF15
MMD
IGFBP4
NONE
FARS2

AKR1C6
IRF9
TSC22D1
ATP6V1H
MPST
RPL10A
BC004004
RMDN2
RNF125
F830016B08RIK
UGT1A6B
SMARCA2
UHRF1BP1L
SEC23B
CTSA
SERPING1
NR1H3
PRPF38B
TMEM56
ABCD3
CTH
SCP2
OSBPL9
CYP2J9
EVI5
SCARB1
GC
DUS1L

PON1
FRMD4B
CARS
FURIN
EED
HP
CNOT7
IFI30
AMFR
TMEM205
SC5D
GCK
ABRACL
2-Mar
2-Mar
BRF1
CFL2
RDH11
FBP1
PAPD4
HMGCR
NDRG2
NABP1
TSTA3
CYP2D37-PS
CYB5R3
PACSIN2
CPPED1

MRPL18
TEDC2
SLC29A1
FECH
CHPT1
HDLBP
CYP2C23
RBM4
BBIP1
HC
MCM10
SLC25A39
ALTERNATIVE 3' SPLICE SITES
GENE
METTL8
0610031O16RIK
TDO2
ST3GAL3
WDTC1
KIF1B
NECAB1
GM21320
MUP17
EVI5
SLC15A4
ASL
GC
GC

SLCO1A4
HP
HP
SLC38A3
CFH
SIDT2
PTS
ODR4
NUP88
FH1
1-Mar
COX16
FAM193B
CAST
DDHD1
NDRG2
HNRNPC
DPYS
NUDCD1
CYP2D37-PS
MASP1
BC1
CREB3L3
CREM
SLC23A1
FGF1
CYP2C67
CYP2C68

METAP2
RRBP1
STAT3
CP
CP
FGG
FGG
PKLR
TXNIP
PANK4
NONE
SDCBP
FGGY
HGFAC
ALB
AFM
MUG2
EVA1A
ARNTL
MOSMO
ATP13A1
A230050P20RIK
SLTM
OTC
C730036E19RIK
IVNS1ABP
SEC16B
SLC35B1

ADK
MAT1A
ZC3H13
PRLR
MYLK
HGD
SON
LUC7L
SAFB
AES
NONE
ABCC2
ENTPD8
GM5424
PRRC2B
NONE
UPP2
CASSETTE EXON EVENTS
GENE
SBF1
SAMHD1
GM14403
ATXN10
SARNP
IP6K2
CHORDC1
STAG2
ZFP207

NUMB
XDH
C6
MRAP
STK38
CYP3A25
NONE
TXNRD1
ACLY
NR1I2
C4A
RPS19
NECAB1
TPRKB
SLC22A30
HPN
MUG2
HCFC1R1
CYP4A12A
RPS24
TRAF7
CES1F
ABAT
CHPT1
CYP2J5
FAM162A
SLC25A47
PON1

CD302
GPD1
DMGDH
GLYAT
PAH
GM5424
HPD
MUG1
SERPINA1E
PDIA4
PECR
LACTB2
CES3A
RRBP1
GOT1
SREBF1
SLC38A2
OSBPL9
GM4952
TANK
ZRANB1
PUM2
LARP4B
HNRNPH1
SNRPA1
NONE
IST1
SMIM20

YTHDF3
HNRNPH3
SDR42E1
KIF21A
NT5C2
EIF4ENIF1
ZFP638
NR2C2AP
TLE1
MARS
RBPMS
TBC1D31
COMPOSITE SPLICING EVENTS
GENE
UOX
TDO2
UBAP2L
RSRP1
H6PD
ERRFI1
NONE
ALDOB
MUP16
NONE
MUP3
MUP3
MUP3
ACOX1

SDS
BAIAP2
QDPR
PCYT2
OCIAD1
ADIPOR2
MUG1
SLCO1B2
RARRES2
SULT2A8
APOE
C4BP
FXYD1
CES3A
CES3A
CES3B
NUDT7
NONE
ACSL1
UBA52
CES1C
SLC38A3
HIGD1A
CFH
TMEM205
PISD-PS3
LAMP2
F9

CLK4
SERPINC1
ALDH3A2
APOA2
NONE
1-Mar
SERPINA1B
SERPINA1C
SERPINA1A
SERPINA1C
SERPINA4-PS1
GM4956
LPIN1
MIA2
SRSF5
SLC17A2
ADK
SHLD2
TMEM19
PCBP2
C9
CYP2D10
SLC38A4
DNM1L
FETUB
KNG1
GM20319
ATP5J

H2-Q10
SAFB2
OSBPL1A
TTR
PROC
GM4951
IIGP1
NONE
SLC22A30
NSMF
